# Lipomatrix: A Novel Ascorbyl Palmitate-Based Lipid Matrix to Enhancing Enteric Absorption of Serenoa Repens Oil

**DOI:** 10.3390/ijms20030669

**Published:** 2019-02-04

**Authors:** Andrea Fratter, Vera Mason, Marzia Pellizzato, Stefano Valier, Arrigo Francesco Giuseppe Cicero, Erik Tedesco, Elisa Meneghetti, Federico Benetti

**Affiliations:** 1 Nutraceutical Research and Innovation Technology Department, Labomar Research, Via Fabio Filzi, 55, 31036 Istrana (TV), Italy; vera.mason@labomar.com (V.M.); marzia.pellizzato@labomar.com (M.P.); 2 Head, Nutraceutical Research and Innovation Technology Department, Labomar Research, 31036 Istrana (TV), Italy; 3 PHF SA, 6900 Lugano, Switzerland; stefano.valier@phfsa.com; 4 Faculty of Medicine and Surgery, Department of Cardiovascular Disease Prevention Research Unit, Internal Medicine and Lipidology, University Alma Mater, Bologna, Via Massarenti 9, Pavilion 2, 40138 Bologna, Italy; afgcicero@gmail.com; 5 ECSIN-European Center for the Sustainable Impact of Nanotechnology, ECAMRICERT SRL, I-45100 Rovigo, Italy; e.tedesco@ecamricert.com (E.T.); e.meneghetti@ecamricert.com (E.M.); f.benetti@ecamricert.com (F.B.)

**Keywords:** ascorbyl palmitate, mono and diglycerides of fatty acids, natural lipophilic compounds, nutraceutical products, *Serenoa repens* oil, enteric bioaccessibility

## Abstract

The class of lipophilic compounds coming from vegetal source represents a perspective in the adjuvant treatment of several human diseases, despite their poor bioavailability in humans. These compounds are generally soluble in fats and poorly soluble in water. The major reason for the poor bioavailability of lipophilic natural compounds after oral uptake in humans is related to their reduced solubility in enteric water-based fluids, leading to an ineffective contact with absorbing epithelium. The main goal to ensure efficacy of such compounds is then creating technological conditions to deliver them into the first enteric tract as hydro-dispersible forms to maximize epithelial absorption. The present work describes and characterizes a new technological matrix (Lipomatrix, Labomar Research, Istrana, TV, Italy) based on a molten fats core in which Ascorbyl Palmitate is embedded, able to deliver lipophilic compounds in a well-dispersed and emulsified form once exposed to duodenal fluids. Authors describe and quantify Lipomatrix delivery of *Serenoa repens* oil through an innovative in vitro model of human gastro-enteric digestion, reporting results of its improved bioaccessibility, enteric absorption and efficacy compared with not formulated *Serenoa repens* oil-containing commercial products using in vitro models of human intestine and prostatic tissue.

## 1. Introduction

Natural compounds with specific reference to plant-derived active ingredients are raising interest as coadjuvant treatment of human diseases. In recent years, this trend has consolidated and many natural products have been introduced into the market under the label of nutritional supplements (NS) or foods with special medical purposes (SMPF). Despite the effective and charming marketing efforts of companies in selling these products following medical routes, the real efficacy of nutraceuticals is still a challenging issue because of the inadequate technological framework for enhancing and testing their bioavailability.

**Figure 1:**
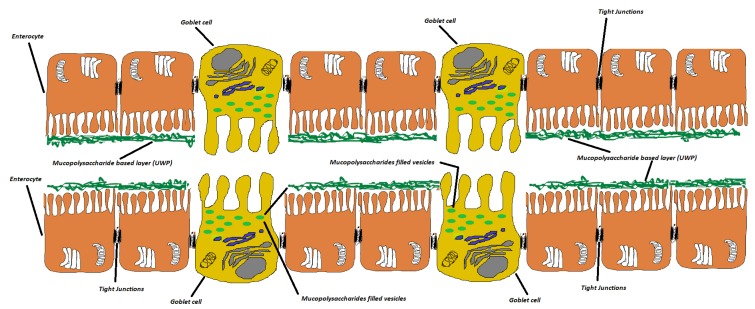
Representation of UWP in the intestine. It is possible to recognize tight junctions and Goblet cells.

Lipophilic active ingredients belonging to the families of terpens, saponins, and vegetal oils are poorly absorbed by intestine mainly because of their low water solubility [1,2]. The interaction of active compounds with intestinal epithelium depends on the dissolution of molecules in water-based intestinal fluids [3]. A viscous mucopolysaccharides rich layer strictly adhering to the enteric epithelium and synthesized by specialized Goblet cells, called unstirred water phase (UWP), (Figure 1) plays a pivotal role in regulating the absorption rate of active ingredients. Mucins, indeed, can interact with poorly-soluble molecules preventing their penetration [4,5].

Only water-soluble molecules or intimately water-dispersed molecules can readily diffuse through this layer, favoring an effective intestinal absorption [6]. According to this premise, lipophilic terpens, such as boswellic acids (BA) extracted from the resin of *boswellia* genus trees, vegetal oils such as ginger oil (GO), *Serenoa repens* (Bartram) Small oil (SRO) and many others structurally similar compounds, are not effectively absorbed through the intestine even in the presence of bile salts [7,8]. Advanced technological matrices and delivery systems able of emulsifying lipophilic compounds or making them hydro-dispersible in the duodenal tract represent promising and effective strategies to improve their bioavailability.

Over the last decades, many companies and research institutes all over the world, tried to discover new performing technologies for enhancing natural compounds bioavailability, in some cases with great success as the patented technology Phytosome^TM^. Phytosome^TM^ consists in a physical complexation of the active ingredient with a phospholipid enabling it to overcoming the enteric barrier, through an enterocyte membrane partitioning mechanism [9,10,11].

Many lipophilic plant-derived active principles have shown several biological relevant actions such as anti-inflammatory activity, enteric physiology restoring activity, and osteoarticular health promoting activity. However, their positive impact on human health is often limited by their poor solubility in aqueous systems, such as digestive fluids, so leading to low enteric bio-accessibility and bioavailability. Among them, SRO is a vegetal oil largely used in clinical practice to reduce symptoms of human prostatic hyperplasia (HPH). This oil that mainly works by inhibiting 5-α-reductase enzyme in prostatic cells and other androgen-sensible cells (sebaceous glands, hair follicles etc.) and by modulating expression of PSA and inflammatory cytokines [12,13] is mainly composed of free fatty acids (80–90% *w*/*w*) and several phytosterols of which β-Sitosterol is the most represented. SRO has shown to be effective in reducing lower urinary tract symptoms (LUTS) such as normalizing urine flow and reducing gland volume [14,15] at dosage of 320 mg of oil titrated at 80–90% in fatty acids per day. Despite this efficacy and the ascertained prostatic tropism [16], not many data are available about its overall bioavailability in humans [16,17] and in particular no data are available about the role of peculiar delivery systems in improving it in humans.

Several technological forms such as nanoemulsions (NE), solid lipid nanoparticles (SLN), and self-emulsifying drug delivery systems (SEDDS) have been developed over the years to deliver insoluble or poorly bio-accessible molecules. On the other side, it must be pointed out, that the most part of natural compounds in form of oils and lipids-based formulations (LF) are mainly composed of triglycerides (TG) in form of emulsion, NE and SLN and for this reason they are partially digested by gastric lipase (GL) that cleaves TG generating free fatty acids (FFA) and diacylglycerols (DAG) [18,19,20]. This underestimated physiological phenomenon can dramatically affect the strategy of delivery of the molecules entrapped into these technological systems [21,22], partially or totally compromising their overall bioavailability.

To design a platform for increasing enteric absorption of lipophilic molecules, some requirements should be considered pivotal to achieve a good pharmaceutical delivery tool: (i) delivery systems able of retaining active ingredients in the stomach and releasing them in the duodenum; (ii) systems able of enforcing bile salts effect in forming fine emulsions or micellar dispersions of lipophilic molecules in the first duodenum; (iii) systems stable to enzymes and peculiar environment of the gastro-enteric tract (iii) systems able of entrapping lipophilic compounds, even in oily form, in a hydro-dispersible solid matrix powder to be easily transformed in capsules or tablets. Taking into consideration the aforementioned requirements, a new technological matrix has been designed and developed to enhance lipophilic compounds enteric absorption. This technological platform, named Lipomatrix (Labomar Research, Istrana, TV, Italy), contains a molten fats core aimed to entrapping lipophilic compounds into a gastric-refractory environment and to emulsify them once exposed to duodenal fluids. This technology is based on a peculiar association of low melting point fats such as mono and diglycerides of fatty acids (MDGFA) commonly used in food industry (E471), and ascorbyl palmitate (ASP), an ester of L-ascorbic acid (AA) and palmitic acid (PA) widely employed in food, pharmaceutical and cosmetic industry as antioxidant agent (E304) [23,24]. The precise chemical features of ASP and its peculiar mechanism of action as emulsifying agent and drugs absorption enhancer are fully described in the Supplementary file section.

In this work, we present for the first time, the technology Lipomatrix and its efficacy in favoring apparent absorption and improving biological activities of SRO through human cell lines models. Beside this main goal, the apparent enteric permeability rate of an extract of Cranberry (Flowens^TM^) and Lycopene vehiculated in the mentioned technological matrix with SRO was assessed. 

## 2. Results and Discussion

### 2.1. Differential Scanning Calorimetry (DSC) Analysis

DSC analysis was performed on MDGFA, mannitol, ASP, SRO, and Lipomatrix as described in Section 3.2.1.2, in order to evaluate possible changes in the crystallinity structure of the single raw material once embedded into the matrix and thereby speculate on its prevalent amorphous or crystalline state. In Supplementary file DSC thermograms for the single substances are reported.

**Figure 2:**
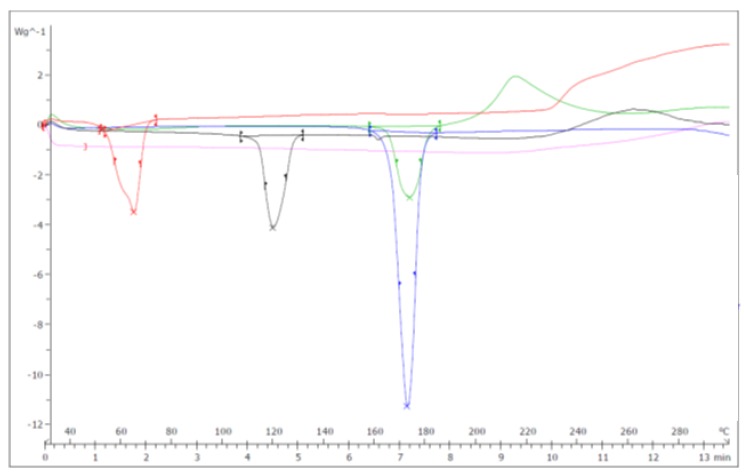
Overlapping thermograms of LIPOMATRIX containing SRO and single components. Black line represents ASP. Red line represents MDGFA. Green line represents Lipomatrix with SRO. Blue line represents Mannitol. Pink line represents SRO.

From the analysis of the Lipomatrix thermogram achieved in the range temperature of 25–125 °C (Supplementary file) it is possible to recognize a melting pick between 47 and 55 °C coherent with the melting point of MDGFA even though there is a significant decrease in the melting temperature pick of about 10 °C in respect to MDGFA alone. This could mean that some interaction phenomena of MDGFA with the other matrix components occurs during Lipomatrix preparation, with an overall increase of the amorphous structure respect to the pure raw material [25,26]. Interestingly, comparing the overlapping thermograms of single components and Lipomatrix one in the range 30–300 °C (Figure 2, Table 1), it is possible to ascertain that both endothermic and exothermic picks of ASP completely disappear, while the only distinctive endothermic pick seems to coincide to Mannitol one. This fact could be explained considering the effective dissolution of ASP into the warmed lipidic matrix, according to the method of production, taking place to a prevalent amorphous or disordered crystalline phase molecular dispersion in the lipid matrix after cooling [27]. Finally, the appearing of a well-shaped exothermic pick in the Lipomatrix sample around 220 °C, not recognized in none of the single components thermograms, could means that a sort of crystallization phenomenon occurs during the warming of the mix of lipophilic components and its further rapid cooling onto the Mannitol to get the final matrix. This phenomenon could be a further proof of an occurring structural change.

### 2.2. In Vitro Assessment of Lipomatrix Containing *SRO* Gastric Resistance

**Figure 3:**
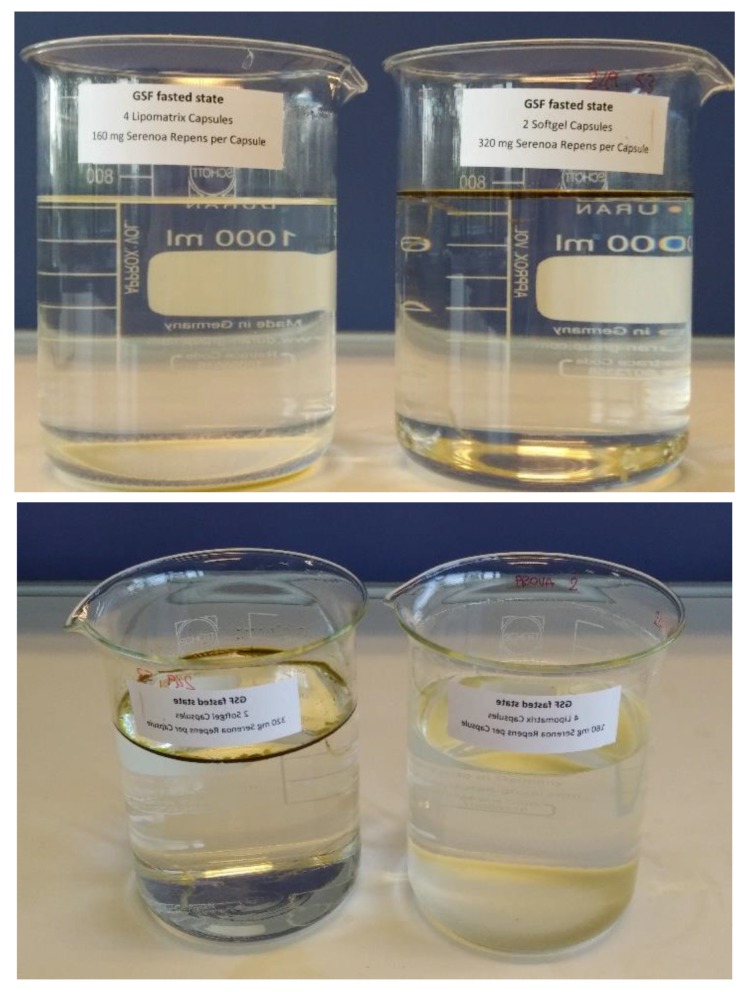
Visual comparison of the liquids coming from disaggregation test in GSF between Lipomatrix containing SRO (left beaker) and soft-gel SRO contained in two soft-gel capsules (right beaker). It is clearly appreciable the presence of an oily layer of SRO in the right beaker relating to the soft-gel capsules and the absence of the oily layer in the surface of the GSF liquid relating to Lipomatrix powder.

One of the three aliquots (triplicate) coming from the gastric-resistance experiment with Lipomatrix powder containing SRO described in Materials and Methods (Section 3.2.4) and the one coming from the same experiment assessed on equivalent amount of not formulated SRO contained in two common gelatin soft-gel capsules have been collected and visually compared each other. An oily layer on the GSF surface is appreciable only in the beaker containing SRO from the soft-gel capsules, while no oily separation is appreciable in the beaker containing Lipomatrix powder. This provides a significant evidence that no SRO release occurred throughout the GSF during the test (Figure 3). 

**Figure 4:**
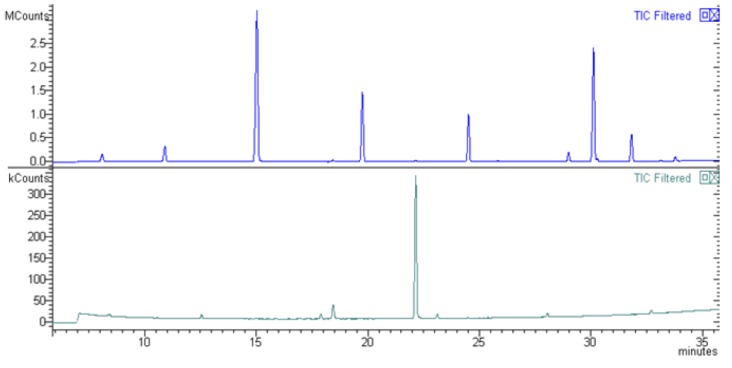
GC-MS overlapping chromatograms of SRO raw material and one of the aliquots of GSF tested (in triplicate, see the supplementary file). The upper graph shows the peculiar picks of fatty acids present in SRO (raw material) that completely disappeared in the lower graph (Lipomatrix specimen), clearly indicating that no SRO release from Lipomatrix has occurred.

GC-MS analysis confirmed the absence of SRO derived fatty acids in GSF with Lipomatrix, suggesting that the technology guarantees a complete gastric resistance of SRO at full therapeutic dosage (Figure 4). The major explanation of this observed behavior is that ASP remains unionized in the stomach since low pH does not permit its enediolic ionization (pH < pKa) and this phenomenon contributes, in synergy with MDGFA, to the overall gastric-resistance of the powder (see the supplemental file section for more detailed explanation).

### 2.3. In Vitro Emulsification 

#### 2.3.1. Emulsifying Behavior in FaSSIF-V2

**Figure 5:**
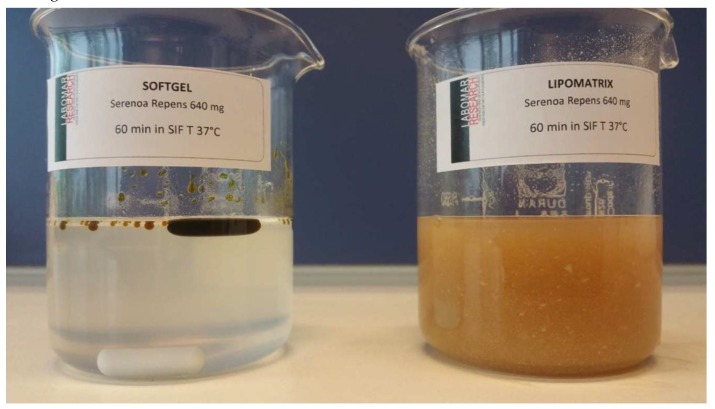
Differential behavior in FaSSIF-V2 at 37 °C, 60 min. of 2 gelatin based soft-gel containing not formulated SRO, 640 mg (left) and Lipomatrix powder containing the same amount of SRO (right). It is possible to recognize the emulsifying capability of Lipomatrix in comparison with soft-gel in which no apparent emulsification occurs. Some insoluble particles suspended in FaSSIF-V2 and ascribable to insoluble excipients such as Magnesium Stearate and amorphous silica are appreciable.

The amount of Lipomatrix powder containing 640 mg of SRO has been dispersed in FaSSIF-V2 at 37 °C under moderate stirring conditions for 1 h, according to the method described in Materials and Methods section (Section 3.2.1.2 and Section 3.2.3). The same experiment has been conducted testing two soft-gel capsules containing the same amount of unformulated SRO. Lipomatrix, in force of the auto-emulsifying profile awarded by the association of MDGFA and ASP at the duodenal simulated conditions, allows a much better emulsification of SRO in FaSSIF-V2, creating the postulated synergy with bile salts that prelude to a better bio-accessibility of the oil to the absorbing epithelium. On the contrary, the soft-gel containing unformulated SRO does not take place to any significant emulsion or micellar dispersion that could be visually recognizable (Figure 5). This experimental evidence confirms the hypothesis of reduced and incomplete emulsifying property of sole bile towards oily active ingredients. 

#### 2.3.2. DLS Analysis of Emulsified Dispersion of Lipomatrix in FaSSIF-V2 

**Figure 6:**
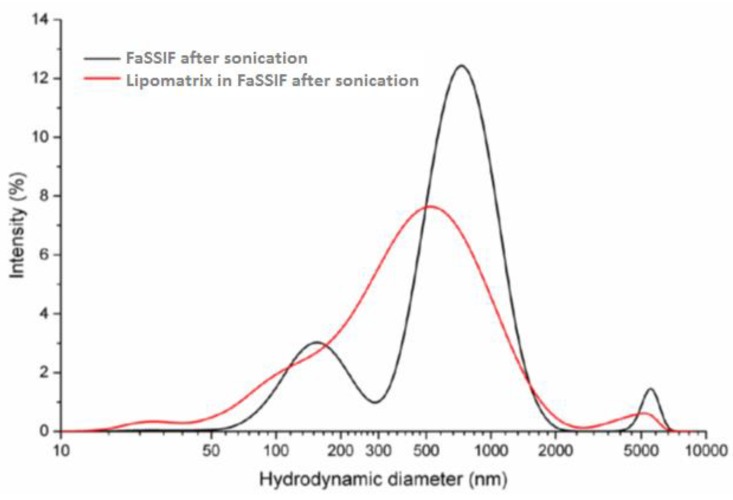
Particle size distribution of FaSSIF-V2 (black Line) and LIPOMATRIX in FaSSIF-V2 (red line) after sonication (*n* = 5).

The DLS analysis of the sample containing SRO entrapped in Lipomatrix and dispersed in FaSSIF-V2 produced the size distribution curve reported in Figure 6 (red line) whose descriptive parameters are reported in Table 2. 

**Figure 7:**
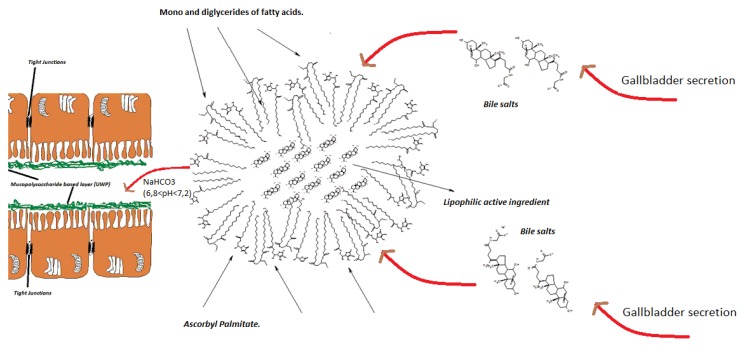
Representation of Lipomatrix mechanism of delivery: lipophilic molecule entrapped in a mixed micellar structure composed of MDGFA, ASP, and bile salts.

The FaSSIF-V2 sample shows a high polydispersity index (0.703) with three well-defined particles populations (769, 161 and 5468 nm) of which the first one is the most abundant and the third the least one. The sample of Lipomatrix dispersed in FaSSIF-V2 shows an elevated polydispersity index (0.529) as well, even though lower than FaSSIF-V2 alone and is composed of three groups of particles (528, 27, and 4511 nm), of which the first one is largely the most represented. The DLS evaluation seems to consolidate the visual hypothesis that Lipomatrix can effectively disperse SRO in FaSSIF-V2, giving rise to an even more finely dispersed system than FaSSIF-V2 alone (lower polydispersion index and presence of a prevalent population around 500 nm). Lipomatrix seems therefore to create a more uniform micellar dispersion in force of the presence of both ionized ASP and MDGFA. ASP becomes partially ionized in the presence of enteric fluids (pH > pKa) and in force of this ionization it behaves as hydro-soluble surfacting agent, capable of emulsifying SRO in synergy with MDGFA and bile salts, generating mixed micellar structures [28,29] (Figure 7). This data gives an analytical confirmation of the visual macroscopic difference of emulsification capability of SRO entrapped in Lipomatrix compared to the same unformulated oil dispersed in FaSSIF-V2 as described in Section 2.3.1. Finally, the comparison of the two specimens shows that a new population ascribable to Lipomatrix, with average hydrodynamic diameter of 27 ± 6 nm, was generated.

### 2.4. *SRO*, PCA, and LYC Bioaccessibility

**Figure 8:**
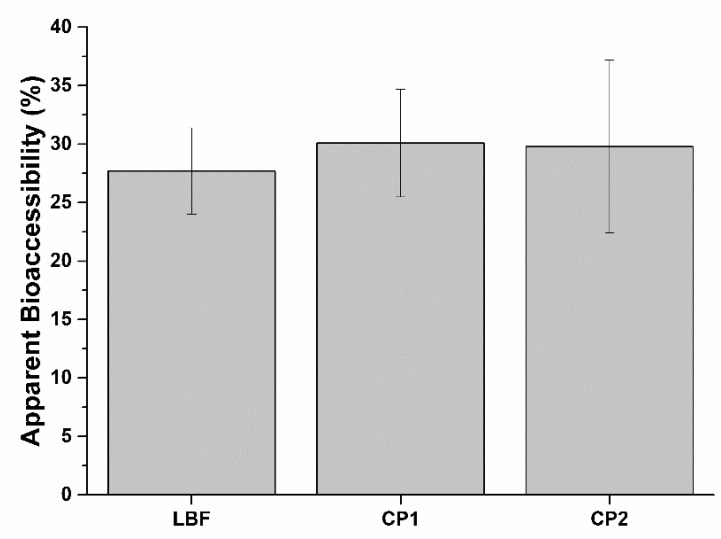
Apparent bioaccessibility of SRO contained in LBF and the two commercial formulations, CF1 and CF2. (*n* = 3).

As previously mentioned, therapeutic applications of active principles from plants, such as SRO oil or curcumine, are hindered by their poor solubility in aqueous medium like the digestive fluids. Although their solubility during the digestive process is slightly improved by the emulsifying activity of bile salts, new technologies aimed at improving lipophilic active principle bioaccessibility are needed. To investigate Lipomatrix-based formulation (LBF) performance compared to the commercial formulations, we exposed a single dose of each formulation to in vitro digestion procedure mimicking human adulthood and evaluated the total amount of active principles and the apparent bioaccessible fraction released from its matrix. The apparent bioaccessible fraction includes the portion available to be absorbed. LBF has similar SRO apparent bioaccessibility of CF1 and CF2 formulations, with a good emulsifying effect of Lipomatrix technology (Table 3 and Figure 8). Lipomatrix emulsifying efficiency is similar to CF2, which contains soy lecithin emulsifier. CF1 has a lower emulsifying efficiency than the others do since it does not contain any specific emulsifier in the formulation. CF1 emulsion is only due to the presence of bile salts in the intestinal compartment. Bile acts, to some extent, as a surfactant, helping to emulsify lipids and lipophilic molecules and increasing their absorption.

In addition to SRO, LBF contains PCA and LYC as active principles. As expected from the gastric resistance test, Lipomatrix technology preserves PCA from degradation, as indicated from their total amount and apparent bioaccessible fraction after digestion (Table 4). Protective effect was also observed on LYC, which is preserved from digestion (Table 4).

### 2.5. Impact of Digested Formulations on Intestinal Epithelium Viability

**Figure 9:**
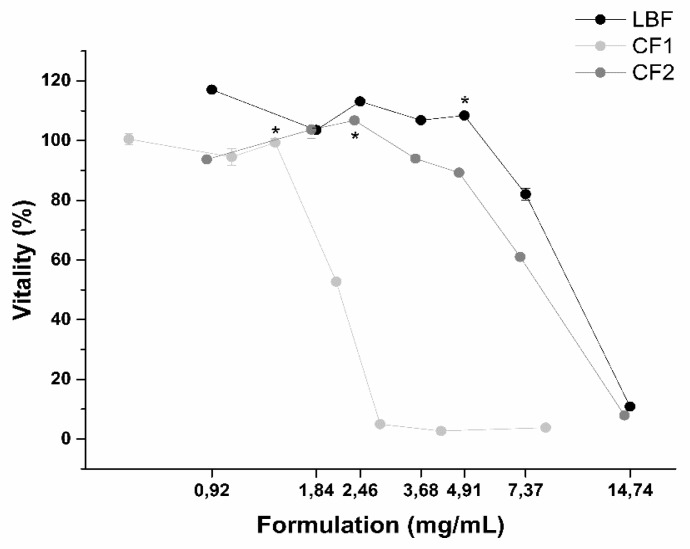
Impact of formulations on intestinal mucosa viability evaluated by determining dose-response curves on formulation concentrations. * Highest non-toxic concentration. (*n* = 3).

Therapeutic formulations must respond to safety criteria. Taking into consideration their dose and posology, formulations should not negatively affect patients. In particular, damages to the intestinal epithelium must be carefully avoided. Before measuring apparent permeability of the three formulations, the impact of digested formulations on intestinal epithelium viability and integrity was assessed. To this aim, intestinal monolayers were exposed to increasing concentrations of the three formulations, and dose-response curves were obtained (Figure 9). From these curves, half maximal effective concentrations (EC50) were calculated (Table 5). As emerged from dose-responses curves and EC50 values, LBF is the safest formulation. 

### 2.6. *SRO*, PCA, and LYC Absorption Rate 

Based on the impact of digested formulations on intestinal epithelium viability and posology (2 capsule/day LBF, 1 capsule/day CF1 and CF2), we set experiments for determining SRO, PCA, and LYC absorption rate. In vitro intestinal epithelia were exposed to the digested formulations for 3 h, and SRO (as fatty acids), PCA and LYC were measured in both apical (lumen) and basolateral (serosal) chambers. Absorption rate was then calculated and expressed as percentage of absorption.

PCA and LYC values in the basolateral compartment were below the detection limits (<0.005 mg/mL and <0.001 mg/mL respectively), as expected considering the low amount and titer of LYC (1.33%) and PCA (2.45%) in LBF. Concerning SRO, its absorption rate is higher in LBF than CF1 and CF2 (Table 6), though this difference is considered to be not statistically significant with respect to CF1. 

**Figure 10:**
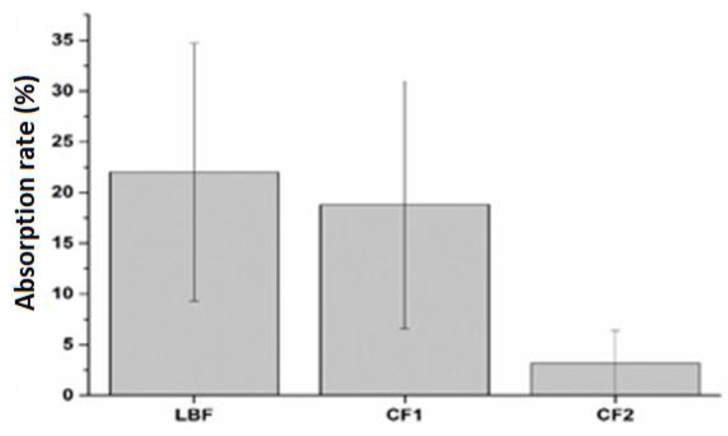
Absorption rate of the three formulations: LBF, CF1, and CF2. (*n* = 3).

Despite apparent bioaccessibility values, the highest absorption rate of LBF suggests that the Lipomatrix technology supports the bioaccessible form of SRO more than the other two formulations (Figure 10).

### 2.7. Impact of Digested Formulations on Intestinal Mucosa Viability and Integrity

**Figure 11:**
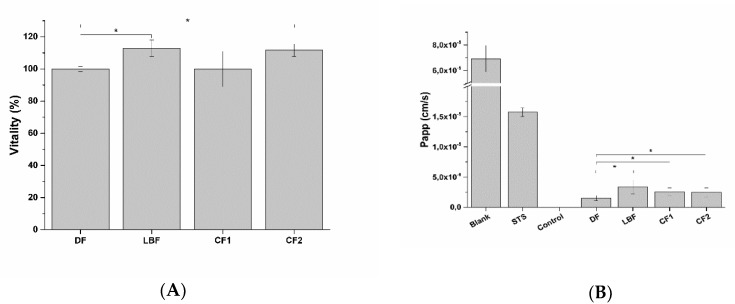
Cell vitality (**A**) and apparent permeability (P_app_) (**B**) of intestinal epithelium exposed to digestive fluids (DF; control) and digested formulations (*n* = 3). LBF is composed of SRO, PAC, and lycopene bioaccessible fractions as reported in Table 5 and Table 6. CF1 and CF2 represent the bioaccessible fractions as reported in Table 3.

After exposure of intestinal epithelia to digested formulations, Caco-2 monolayer viability and barrier integrity were analyzed. As expected, no viability reduction was observed during absorption rate experiments, while a slight but significant increase in apparent permeability (P_app_) was observed for all the three digested formulations (Figure 11).

**Figure 12:**
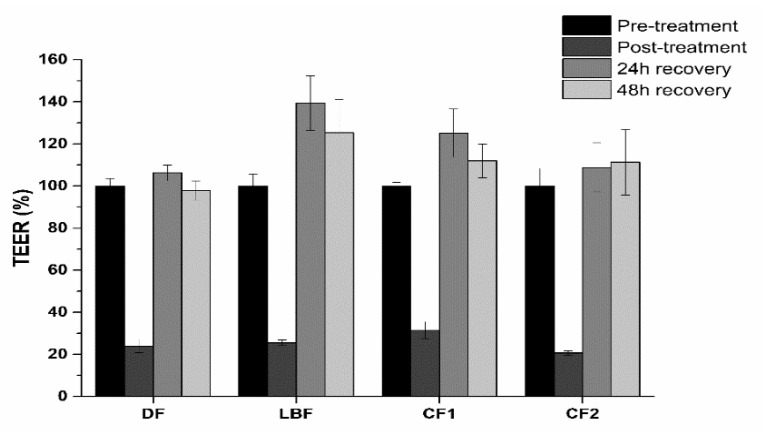
TEER values recorded before the treatment (Pre-treatment), after exposure to digested fluids (DF) or formulations (Post-treatment) at the bioaccessible concentrations, and upon 24 and 48 h recovery. Values are expressed as percentage of the pre-treatment TEER value (*n* = 3).

Increase of the absorption rate parallels to a reduction of intestinal epithelia trans-epithelial electrical resistance (TEER) after exposure (Figure 12). Both digested formulations and digestive fluids reduced TEER transiently, since they fully recovered to pre-treatment values.

### 2.8. Cytotoxic Effect of Diclofenac and Permeable Fractions on Prostatic Epithelium Model

SRO is one of the most popular natural treatments for treating HPH as mentioned. The permeable fractions were tested on prostatic epithelium in vitro model to evaluate their efficacy against inflammation process. Before performing efficacy tests, cytotoxicity of the anti-inflammatory positive control Diclofenac and bioaccessible fractions on LNCaP was measured.

**Figure 13:**
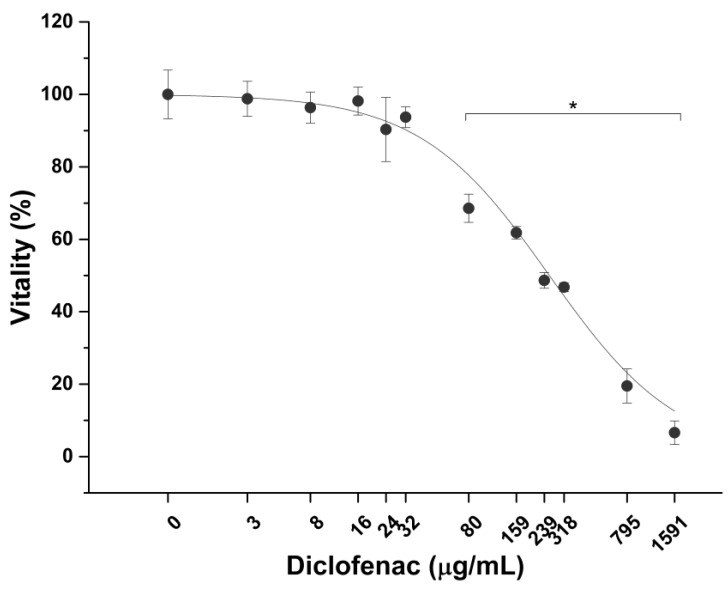
Effect of diclofenac on LNCaP prostatic cells vitality. * *p* < 0.05 (*n* = 3).

**Figure 14:**
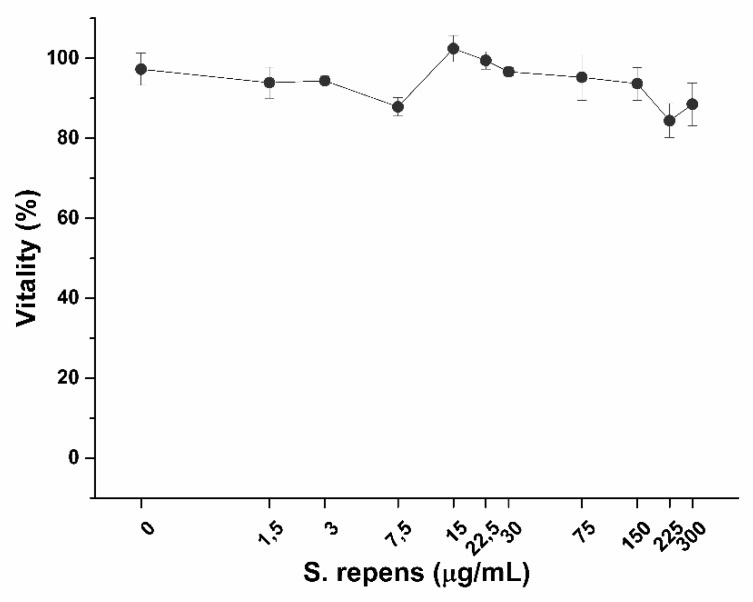
Effect on LNCaP prostatic cells vitality after 6 h exposure to SRO. (*n* = 3).

As shown in Figure 13, diclofenac significantly decreases prostatic cell viability at a concentration of 80 µg/mL after 6 h exposure, with a highest non-toxic concentration of 32 µg/mL. Conversely, no effect on cell vitality was observed on LNCaP cells after treatment with bioaccessible fractions (Figure 14).

### 2.9. Prostate-Specific Anti-Inflammatory Activity of Permeable Fractions

In recent years, inflammation has been recognized as the main phenomenon responsible for the onset of HPH, a noncancerous increase in size of the prostate, leading to the appearance of bothersome symptoms, such as frequent urination, difficult urination, weak stream, inability to urinate, and loss of bladder control. Prostatic-specific anti-inflammatory activity of bioaccessible fractions was evaluated on the in vitro prostatic epithelium model based on LNCaP cells, by measuring expression levels of pro-inflammatory cytokines IL-1β and TNF-α.

**Figure 15:**
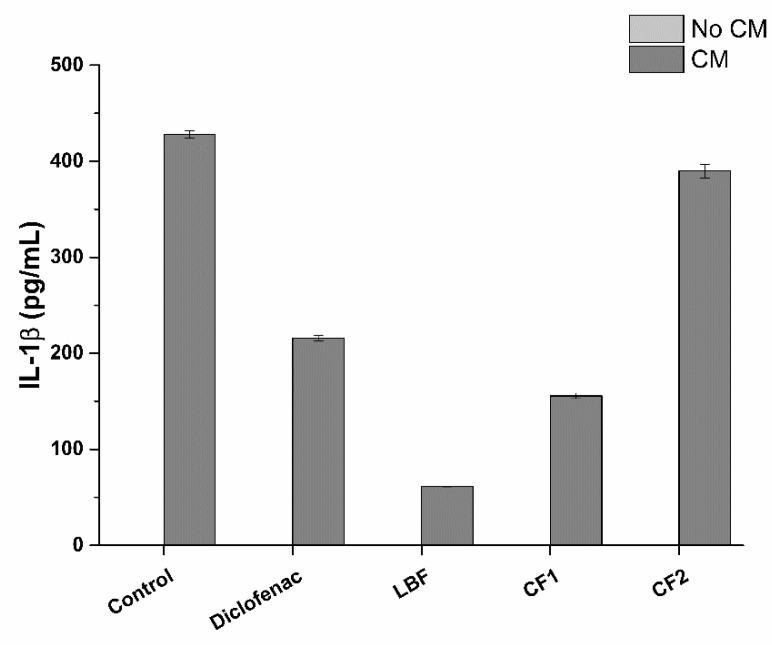
Production profile of IL-1β following treatment of LNCaP prostatic cells with bioavailable fractions: LBF: 22% SRO; CF1: 18.8% SRO; CF2: 3.2% SRO (Table 6). Diclofenac was used at 32 µg/mL. CM: Conditioned medium. * *p* < 0.05 (*n* = 3).

As shown in Figure 15, bioaccessible fractions significantly reduce the production of IL-1β compared to the control. In particular, the higher amount of SRO of LBF compared to the two commercially available formulations, presents the strongest effect, with an 85% reduction in IL-1β production.

**Figure 16:**
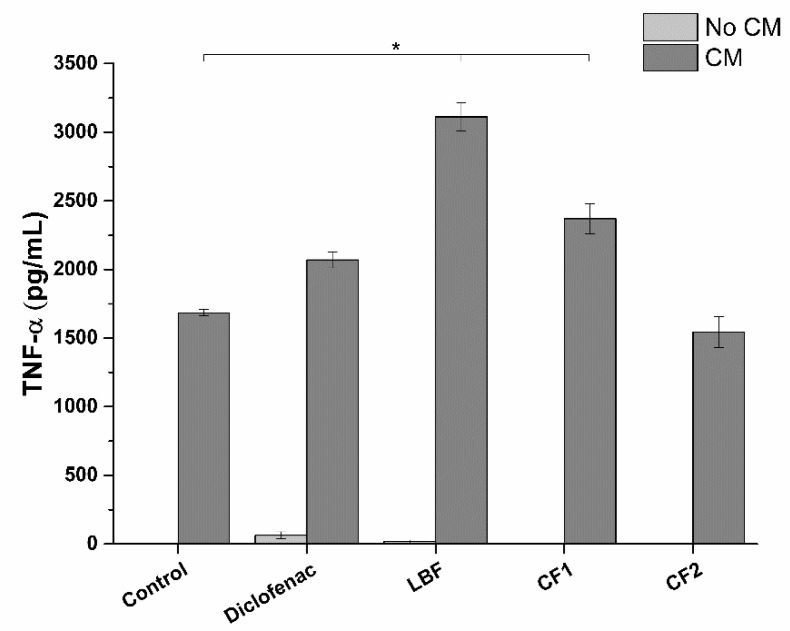
Production profile of TNF-α following treatment of LNCaP prostatic cells with bioavailable fractions: LBF: 22% SRO; CF1: 18.8% SRO; CF2: 3.2% SRO (Table 6). Diclofenac was used at 32 µg/mL. CM: Conditioned medium. * *p* < 0.05 (*n* = 3).

Interestingly, no TNF-α reduction was observed for the other considered pro-inflammatory cytokine, TNF-α (Figure 16).

**Figure 17:**
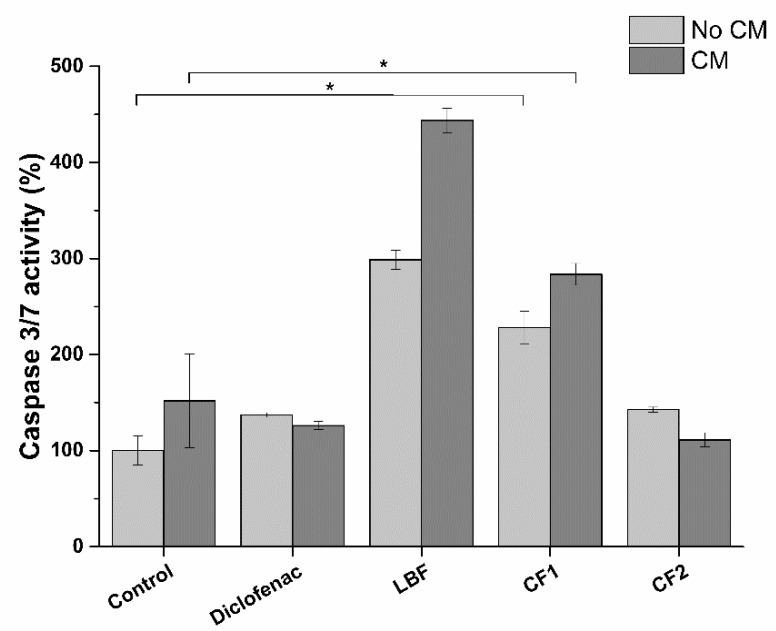
Pro-apoptotic activity of bioavailable fractions on LNCaP prostatic cells: LBF: 22% SRO; CF1: 18.8% SRO; CF2: 3.2% SRO (Table 6). Diclofenac was used at 32 µg/mL. CM: Conditioned medium. Samples are normalized on no CM control. * *p* < 0.05 (*n* = 3).

Conversely, to IL-1β, the production of TNF-α significantly increases upon exposure to LBF and CF1 bioaccessible fractions. No effect was observed for CF2. This peculiar trend could be explained considering the pro-apoptotic activity of SRO. Indeed, TNF-α is a cytokine known to be involved in the apoptotic process. Silvestri and colleagues [30] demonstrated that SRO extract induces apoptosis in LNCaP cells. To test this hypothesis, we evaluated the pro-apoptotic effect of SRO absorbable fractions on the prostatic epithelium model in both uninflamed and inflamed conditions (Figure 17).

**Figure 18:**
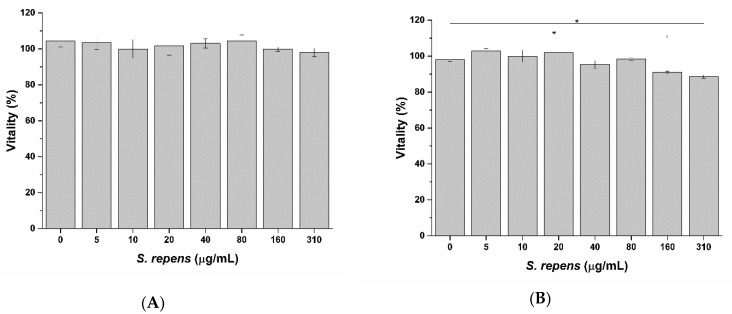
Effect on LNCaP prostatic cells vitality after 6 h (**A**) and 24 h (**B**) exposure to SRO. * *p* < 0.05 (*n* = 3).

As expected, pro-apoptotic activity induced by bioaccessible fractions well correlates with TNF-α expression, meaning a role of SRO in inducing apoptosis against inflamed and tumor prostatic cells. The apparent contrast between LNCaP prostatic cells vitality (Figure 14) and apoptosis (Figure 17) results could be explained by an early-apoptosis phenomenon. During early apoptosis phenomenon, indeed, cells retain their vitality. Activation of early apoptosis cascade leads to cell dead at later times, suggesting a decrease in LNCaP prostatic cells viability following prolonged exposure to SRO. While no effects were observed after 6 h incubation (Figure 18A), cell viability significantly decreases after 24 h exposure (Figure 18B). 

### 2.10. Activity of Bioaccessible Fractions on PSA Secretion

**Figure 19:**
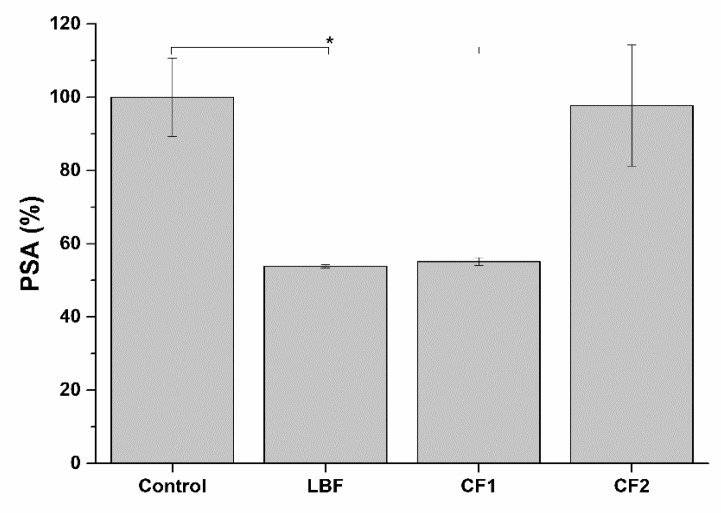
PSA secretion in LNCaP prostatic cells treated with bio-accessible fraction of the different formulations. * *p* < 0.05 (*n* = 3).

Prostate-Specific Antigen (PSA) is considered the main serum marker for the progression of prostate cancer [31]. A decrease in PSA secretion, following treatment with bioaccessible fractions, indicates a potential therapeutic effect of the formulations. To verify the effect of the bioaccessible formulations on PSA secretion, LNCaP hormone-sensitive cell line was used. Compared to control conditions, PSA secretion decreased in cells treated with LBF and CF1 bioaccessible fractions, while no differences were observed in cells treated with CF2 formulation (Figure 19). 

### 2.11. Smooth Muscle Myorelaxing Activity

The decrease in epithelial-to-stromatic tissue ratio is a well-known marker of benign prostatic hypeplasia (BPH) development. In the prostate, the stromal component is mainly composed of smooth muscle tissue, which is normally contracted in response to adrenergic stimulation. As a result, urethra lumen is reduced and urination made difficult. To explore the potential effect of tested formulations on this BPH symptom, the myorelaxing activity of the bioaccessible fractions on smooth muscles was evaluated using WPMY-1 myofibroblast in vitro model.

**Figure 20:**
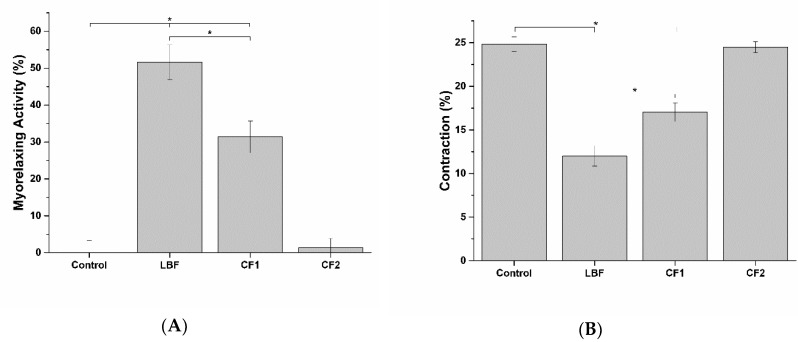
Myorelaxing (**A**) and contraction inhibitory (**B**) activity of the bioaccessible fraction of the different formulations on an in vitro model of smooth muscle. * *p* < 0.05 (*n* = 3).

As shown in Figure 20, no muscle relaxation was induced by CF2 bioaccessible fraction. Conversely, the bioaccessible fractions of LBF and CF1 showed a significant myorelaxing activity on smooth muscles, with LBF presenting the highest myorelaxing activity.

## 3. Materials and Methods

### 3.1. Materials

*Serenoa repens* oil 85% fatty acids GC was purchased from Naturex S.p.a. (Caronno Pertusella, VA, Italy). Ascorbyl palmitate and lecithin were purchased from A.C.E.F. (Fiorenzuola D’Arda, PC, Italy). Mono- and diglycerides of fatty acids were from BASF Italia S.p.a. (Cesano Maderno, MB, Italy). Mannitol, synthetic amorphous silica, magnesium stearate and sodium bicarbonate were purchased from Giusto Faravelli S.p.a. (Milano, Italy). Sodium chloride was purchased from Fagron Italia S.r.l. (Quarto Inferiore, BO, Italy). Thaurocholic acid was purchased from Shangai T and W Pharmaceuticals Co. (Shangai, China), maleic acid and lipase from porcine pancreas were purchased from Sigma-Aldrich (St Louis, MO, USA), Flowens^TM^ and lycopene (powder titrated at min. 6% *w*/*w*) were purchased from Naturex.

Caco-2 human colon adenocarcinoma cell line (ATCC^®^ HTB-37™), LNCaP androgen-sensitive human prostate adenocarcinoma cell line (ATCC^®^ CRL-1740™), WPMY-1 human myofibroblast stromal cell line (ATCC^®^ CRL-2854™) and THP-1 (ATCC^®^ TIB-202™) were purchased from ATCC (Manassas, VA, USA). High glucose Dulbecco’s Modified Eagle Medium (DMEM), Roswell Park Memorial Institute (RPMI) 1640 Medium, Hanks’ Balanced Salt Saline (HBSS), non-essential amino acids (NEAA), L-glutamine, penicillin-streptomycin mix, lipolysaccharide (LPS), diclofenac, dihydrotestosterone (DHT), phorbol 12-myristate 13-acetate (PMA), proantocyanidine A, B1, and B2 standards and Lucifer Yellow (LY) were purchased from Sigma-Aldrich (St Louis, MO, USA). Foetal bovine serum (FBS) was purchased from Euroclone (Milan, IT). Interleukin 1β (IL-1β), Tumor Necrosis Factor α (TNF-α) and prostate-specific antigen (PSA) ELISA kit were purchased was purchased from R and D Systems, PeproTech (London, UK) and Abcam (Cambridge, UK), respectively. Cell contraction assay was purchased from Cell Biolabs (San Diego, CA, USA). Transwell^®^ insert were purchased from Millipore (Burlington, MA, USA). CellTiter 96^®^ AQueous One Solution Cell Proliferation Assay (MTS) and Apo-ONE^®^ Homogeneous Caspase-3/7 Assay were purchased from Promega (Madison, WI, USA). C18 cromatographic columns were purchased from Agilent (Santa Clara, CA, USA). Cyclo-oxygenase (COX) activity assay kit was purchased from Cayman Chemicals (Ann Arbor, MI, USA).

### 3.2. Methods

#### 3.2.1. Preparation of Lipomatrix Powder 

##### 3.2.1.1. Preparation of Lipomatrix Powder with SRO, Flowens, and Lycopene

MDGFA and ASP were dry mixed in a beaker and the mixture was melted at temperature of 80 °C under mechanical stirring (IKA^®^ RTC Basic Staufen, Germany). The resulting oily liquid phase was added of SRO 85% fatty acids GC and Lycopene (powder titrated at min 6% *w*/*w*), maintaining the temperature at 75 °C. Mannitol and Flowens^TM^, previously mixed and cooled down in fridge at 15 °C, were put in a planetary mixer (Kenwood KMX750RD, De Longhi Treviso, Italia, Kenwood group) and the hot oily phase was added on it in small continuous additions under mixing so to induce an instant and uniform solidification of the lipid phase onto the cold powder. The formed composite powder was cooled down at room temperature for 24 h. Finally, the granules were passed through a 1.5 mm mesh net connected to the mechanical sieve (Vasquali, Marchesini group, Cerro Maggiore (MI), ITALY). The resulting powder was added of synthetic amorphous silica and magnesium stearate to improve powder flowability. Type 0 animal gelatin capsules were filled with the powder using a manual encapsulator (MultiGel) so that the content of any capsule corresponded to 160 mg of SRO, 125 mg of Flowens and 5 mg of lycopene (powder titrated at 6% *w*/*w*).

##### 3.2.1.2. Preparation of Lipomatrix Powder with SRO Alone

The same method of Section 3.2.1.1. in which no Flowens^TM^ and lycopene powder have been introduced.

#### 3.2.2. Flow Property

Flow through an orifice was measured according to Ph. Eur. Chapter 2.9.36. The test was performed using a metal truncated cone Flowability Tester (Flowability Tester BEP Auto Copley Scientific, Colwick, Nottingham) with different size of the orifice. The time it took for 100 g of powder to pass through 25–15–10 mm diameter orifices was measured in triplicate. 

#### 3.2.3. In Vitro Emulsification Test

In vitro emulsification in FaSSIF-V2 was performed as follows: a 100 mL glass beaker was filled with 50 mL of simulated gastric fluid (GSF, according to Eur. Pharmcopea) and placed at 37 °C ± 0.5 °C (IKA^®^ RTC Basic). FaSSIF-V2 was chosen as model of enteric fluid to assess the real capability of Lipomatrix to create soluble micellar forms in the duodenum without any interfering molecules coming from ingested foods such as higher Lecithin concentration than FaSSIF-V2 and oleic acid (OA) esters and salts (FeSSIF-V2) [32]. GSF was realized from demineralized water (inverse osmosis process) added of dilute solution of HCl up to pH = 1. The quantity of Lipomatrix powder corresponding to 4 capsules as described in Section 3.2.1.2. containing a total amount of 640 mg of SRO, has been dispersed in the afore described GSF at 37 °C under moderate magnetic stirring (200 rpm, IKA^®^ RTC Basic) for 90 min simulating gastric transit time. After that, the suspended Lipomatrix powder was decanted, the GSF was removed and replaced with 50 mL of FaSSIF-V2. FaSSIF-V2, composition was the following: sodium chloride 68.62 mM, thaurocholic acid 3 mM, lecithin 0.2 mM, maleic acid 19.12 mM, lipase from porcine pancreas 100 units/mL, pH 7.20 adjusted with sodium bicarbonate. We preferred to use sodium bicarbonate instead of phosphate buffer, as described in the consolidated FaSSIF-V2 model [33], to simulate the secretion of sodium bicarbonate by pancreas [34]. This model showed furthermore to be useful to verify the pH lowering behavior of Lipomatrix ascribable to ASP ionization, so confirming the postulated mechanism of action. The temperature of the media was set at 37 ± 0.5 °C by means of a thermostatic probe. The powder was maintained in the dissolution medium for 60 min under magnetic stirring (200 rpm, IKA^®^ RTC Basic). During the test, pH probe was introduced in the FaSSIF-V2 dispersion and pH was monitored continuously (Sension + PH3, Hach). Since ASP embedded in Lipoamtrix ionizes in FaSSIF-V2 (pKa < pH), ionization leads to a pH reduction so each 15 min pH was adjusted with sodium bicarbonate to maintain pH not less than 7.00 and simulate the continuous pancreatic secretion of sodium bicarbonate. The test has been assessed with 50 mL of both GSF and FaSSIF-V2 to set the models close to the average volumes, of physiologic fasted condition of gastro-enteric tract in humans [35,36]. 

#### 3.2.4. In Vitro Assessment of Lipomatrix Containing SRO Gastric Resistance (the Test on Lipomatrix Specimens was Performed in Triplicate)

A Semi-Automatic Disintegration Tester (Charles Ischi AG DISI-M) was used to simulate gastric transit of Lipomatix powder as described in Section 3.2.1.2. The dissolution medium was 800 mL of GSF at 37 °C (according to Eur. Ph). Three aliquots of Lipomatrix powder as described in Section 3.2.1.2 containing a total amount of 640 mg of SRO was maintained for 90 min in GSF anyone placed in a single chamber of the mentioned device.

#### 3.2.5. Analytical Characterization

##### 3.2.5.1. Differential Scanning Calorimetry (DSC) Analysis

DSC scans were performed by a Mettler-Toledo DSC calorimeter using the following analysis conditions: 25.00–300.00 °C, 20K/min and 25.00–125.00 °C, 10K/min. The Lipomatrix powder with SRO alone (as described in Section 3.2.1.2) has been processed and each single excipient of which it is composed as well. The main scope of this analysis is to understand the thermal behavior of Lipomatrix powder and if any change in the structure occurs during fusion of the fatty phase and its further rapid cooling during absorption onto the polyol-based powder. The thermal exchanges profile of any component and of the final powder have been determined. 

##### 3.2.5.2. Gas Chromatography-Mass Spectrometry (GC-MS) Analysis on Gastric-Resistance Test Samples

To proceed with GC-MS assay, 5 mL of liquid coming from gastric resistance test chamber were added of 100 µL of metyl pentadecanoate solution (115 μg/mL) as internal standard. Extraction with ethyl acetate was performed three times. The obtained organic phases were pooled together, anhydrified with sodium sulfate and led to dryness. The extracts were added of 3 mL of methanol and 1 mL of dichloromethane and were acidified with concentrated sulfuric acid. Samples were heated under reflux for 40 min, then the solution were cooled down and diluted with water and diethyl ether. The etheric phase was investigated.

The standard was prepared mixing 100 mg of SRO fatty acids GC with 1 mL of metyl pentadecanoate solution (115 μg/mL) as internal standard, and adding 2 mL of dichloromethane, 10 mL of methanol and 0.2 mL of concentrated sulfuric acid. Standard sample was heated under reflux, then cooled down and diluted with water and diethyl ether. The etheric phase was investigated.

The GC-MS system was comprised of a Varian 3800 equipped with autosampler and coupled with a Varian Saturn 2100 MS/MS ion trap mass spectrometer. A HP-88 column (60 m × 0.25 mm) was used for separation (J and W Scientific, Agilent technologies Inc., Santa Clara, CA 95051, USA)

##### 3.2.5.3. Dynamic Light Scattering (DLS) Analysis of Lipomatrix Dispersion in FaSSIF-V2

DLS was performed on emulsified dispersion of Lipomatrix in *FaSSIF-V2* as described in Section 3.2.1.2, corresponding to 640 mg of SRO resulting from in vitro emulsification test described in Section 3.2.3. The size distribution of the particles was evaluated as a signal intensity function only. The conversion of the signal intensity distribution into particles volume or number distribution can cause error propagation since it requires some unavailable parameters (e.g., the particles refractive index). The liquid samples (FaSSIF-V2 and Lipomatrix dispersion in FaSSIF-V2) were analyzed by DLS (Zetasizer Nano S, Malvern). 

#### 3.2.6. Cell Cultures

##### 3.2.6.1. Caco-2 Cell Culture

The Caco-2 human colon adenocarcinoma cells (passage 32 to 42) were seeded in adhesion flask at a density of 2 × 10^3^ cell/cm^2^ in Caco-2 Complete Medium (CCM) (high glucose DMEM, 10% heat inactivated FBS (FBS), 1% non-essential amino acids, 4 mM L-glutamine, and 1% penicillin-streptomycin mix) and cultured at 37 °C and 5% CO_2_ in a humidified incubator. Cell were seeded at 2000 cells/cm^2^ and subcultivated by tryspinization every 7 d when 80–90% confluent. The medium was refreshed every other day.

##### 3.2.6.2. LNCaP Cell Culture

The human prostate cancer cell line LNCaP cells (passage 25 to 40) were maintained in RPMI-1640 medium supplemented with 10% FBS and 1% penicillin-streptomycin mix. The cells were grown at 37 °C in a humidified atmosphere with 5% CO_2_. Cell were seeded at 10,000 cell/cm^2^ and medium changes every other day. As for Caco-2, cells were subcultivated by tryspinization every 7 d when 80–90% confluent. For vitality and anti-inflammatory experiments, LNCaP were seeded 96-well plates and six-well plates, respectively, at a density of 1 × 10^5^ cell/cm^2^ and allowed to adhere for two days prior to experiments, while for prostate-specific antigen (PSA) experiment cells were seeded at a density of 50,000 cells/cm^2^ in 24-well plates.

##### 3.2.6.3. THP-1 Cell Culture

Human THP-1 monocytes (passage was maintained in RPMI-1640 medium with glutamate supplemented with 10% FBS, 100 U/mL penicillin and 100 μg/mL streptomycin (GIBCO, Winsford CW7 3GA, UK). Cells were cultured at a density of 5 × 10^5^ cells/mL in 5% CO_2_ humidified atmosphere at 37 °C and subcultured twice a week. Macrophage differentiation was induced by incubation with 500 nM phorbol myristate acetate (PMA; Sigma-Aldrich, MO, USA) for 24 h. Culture medium was then replaced and cells cultured for an additional 24 h. For medium conditioning, 6 × 10^6^ cell were seeded in 75 cm^2^ flask, differentiate into macrophages as described before and treated with 1 ng/mL LPS for 6 h. At the end of the LPS treatment, medium was recovered and stored at −80 °C until use.

##### 3.2.6.4. WPMY-1 Cell Culture

Human prostate stromal (myofibroblast) (WPMY-1) (passage 40 to 50) were cultured in DMEM, supplemented with 10 % fetal bovine serum (FBS) and 1 % penicillin-streptomycin mix. As for Caco-2, cells were seeded at 2000 cells/cm^2^ and subcultivated by tryspinization every 7 d when 80–90% confluent. The medium was refreshed every other day. For cell contraction experiments, 1 × 10^6^ cells were loaded into each collagen gel.

#### 3.2.7. Determination of Active Principle Absorption Rate

To evaluate the effectiveness of Lipomatrix technology in increasing the enteric absorption rate of lipophilic active principles, we compared the performance of a Lipomatrix-based formulation (LBF) as described in *3.2.1.1* against two commercial formulations, as indicated in Table 7.

##### 3.2.7.1. Digestion Process

A single dose of each formulation listed in Table 7 was exposed to in vitro digestion process simulating the physiological human digestion in the oral, gastric and intestinal compartments. Briefly, the formulations were incubated for 5 min in saliva at 37 ± 1 °C, rotating head-over-heels at 55 rpm, simulating peristaltic movements. Subsequently, gastric juice (pH 1.3 ± 0.1) was added to the mixture and the pH of the sample was checked and, if necessary, adjusted to 2.5 ± 0.5 with NaOH (1 M) or HCl (37% *w*/*w*). The sample was further incubated rotating at 37 °C for 2 h. Subsequently, duodenal juice (pH 8.1 ± 0.1), bile (pH 8.2 ± 0.1) and sodium bicarbonate were added. The pH of this mixture was set at 6.5 ± 0.5 with NaOH (1 M) or HCl (37%) and it was rotated head-over-heels for another 2 h. For simulated digestive fluids composition refer to Walczak et al., 2013. Once completed the digestion process, SRO bioaccessibility was determined by measuring fatty acids with GC/MS, while proanthocyanidin (PAC) and lycopene loaded in Lipomatrix formulation were determined by high pressure liquid chromatography (HPLC).

##### 3.2.7.2. HPLC Analysis of LYC and PCA

Bioaccessible fractions of LYC and PAC, released during the digestive process and absorbed at the intestinal epithelium level, were determined by HPLC. Reversed phase HPLC with absorbance detection (at 475 nm) based on the modified method of Thadikamala et al. (2009) was used for analyzing LYC. HPLC separation was carried out using a Varian Prostar 210 pump system (Agilent) and a UV–VIS detector (Variant Prostar, Agilent), operated at 25 °C. A C18 column (4.6 × 150 mm, Agilent) was used with methanol-acetonitrile-methanol-tetrahydrofuran (THF) (70:25:5, *v*/*v*) as an isocratic eluent. Each sample was dissolved first in methanol-THF (50:50, %*v*/*v*) and diluted as needed in the same solvent. Quantification of the eluted LYC was accomplished by the peak area method using the calibration range of 15 to 150 ppm of LYC (Sigma-Aldrich, Milan, Italy) as external standards. For PACs, HPLC analysis was performed with Variant Prostar HPLC (Agilent) with the same pump system and UV–VIS detector described above. The phenolic compounds were detected at 280 nm with a flow rate of 1 mL/min. The column was operated at a temperature of 25 °C. Separations were carried out with a C18 column (Agilent) in a dual pumping system by varying the proportion of 2.5% (*v*/*v*) acetic acid in water (mobile phase A) and 70% methanol in water (mobile phase B). The solvent gradient elution program was as follows: 10% to 26% B (*v*/*v*) in 10 min, to 70% B at 20 min and finally to 90% B at 25 to 31 min. The injection volume for all samples was 100 μL. The phenolic compounds were analyzed by matching the retention time and their spectral characteristics against those of standards PAC A, B1, or B2 (20 to 400 ppm).

#### 3.2.8. In Vitro Model of Human Intestinal Epithelium

Permeability and absorption rate of SRO (i.e., fatty acids), PAC and LYC were determined using an in vitro model of human intestinal epithelium based on Caco-2 cells. Briefly, Caco-2 cells are seeded on Transwell^®^ polytetrafluoroethylene inserts (1 µm pore size) at an initial density of 1.5 × 10^5^ cells/cm^2^ and allowed to mature and differentiate for 21 days. Indeed, thanks to the compartmentalized nature of the Transwell^®^ system (apical (or lumen) and basolateral (or serosal) compartment), Caco-2 cells differentiate, acquiring morphological and functional feature typical of enterocytes, as the presence of microvilli, tight junctions and P-glycoprotein. Absorption experiment were performed between 21 and 28 days post seeding.

##### 3.2.8.1. Evaluation of the Impact of Digested Formulations on Intestinal Epithelium Viability

To evaluate the impact of digested formulations on intestinal epithelium viability, digested formulations were serially diluted in digestive fluids (from 1:2 up to 1:32 dilution) and added to the apical side of the in vitro intestinal epithelia, while HBSS buffer was placed in the basolateral compartment. Digestive fluids (without formulations) were added to the apical side of the in vitro intestinal epithelia as a negative control. After 3 h incubation, monolayers were washed twice with pre-warmed HBSS and viability of intestinal epithelia was evaluated with MTS assay, according to manufacturer’s instructions. This assay is based on MTS tetrazolium compound reduction by viable cells to generate a colored formazan product that can be quantified by measuring the absorbance at 490 nm. The color intensity at 490 nm was determined with a microplate reader (Synergy4, Biotek, Colonia Santo Domingo Azcapotzalco Distrito Federal, México). Cell viability (%) was expressed as the ratio of the color intensity in the treated groups to that in the control (untreated) group. Absorption rate experiments were performed using non-toxic concentrations determined by dose-response curves.

##### 3.2.8.2. Evaluation of SRO (Fatty Acids), PCA, and LYC Enteric Absorption Rate

Based on dose-response curve information and their posology, digested formulations were added to the apical side of the in vitro intestinal epithelium, while HBSS buffer supplemented with 1% BSA was placed in the basolateral compartment. Due to the lipophilicity of formulation active components, 1% BSA was added to the basolateral compartment for improving their absorption rate. According to the literature (Fossati et al., 2008) [37] the addition of BSA improves the correlation between absorption occurring in Caco-2 cell monolayer and humans. After 3 h incubation, apical and basolateral solutions were collected and fatty acids, proanthocyanidin and lycopene content was determined by GC/MS and HPLC respectively. Absorption rate of SRO fatty acids, PCA and LYC is expressed as percentage of absorption, derived from three independent experiments.

##### 3.2.8.3. Barrier Integrity and Cell Viability

After exposure to digested formulations, cell viability and barrier integrity of the intestinal epithelium model were evaluated. Briefly, at the end of the incubation with the digested formulations, the in vitro intestinal epithelia were washed twice with pre-warmed HBSS and equilibrated in the same buffer for 30 min. Once equilibrated, epithelia barrier integrity was evaluated by measuring the trans-epithelial electrical resistance (TEER) of the cell monolayer with an ERS2 Voltohmmeter (Millipore), equipped with a chopstick electrode. Intestinal epithelium model paracellular permeability was determined with Lucifer Yellow (LY), a fluorescent polar tracer unable to pass through intact tight junctions. Paracellular permeability was measured by adding 0.5 mL of 100 µg/mL LY in HBSS in the apical compartment and 1.5 mL of HBSS in the basolateral compartment. After 1-h incubation, the basolateral fractions were collected and their fluorescence measured with a spectrofluorometer (Synergy 4, Biotek). Apparent permeability coefficient (P_app_, cm/s) was calculated with the following formula:

P_app_ = (ΔC · V)/(Δt · A · C_0_) (1)

where ΔC/Δt is the flow of the molecule being transported across the monolayer during the incubation time (mM/s), V is the volume of the basolateral compartment (cm^3^), A is the area of the membrane (cm^2^), C_0_ is the initial concentration of the molecule in the apical compartment. Finally, cell viability was evaluated by using MTS assay as described above. 

#### 3.2.9. Prostate-Specific Anti-Inflammatory Activity

The prostate-specific anti-inflammatory activity of the bioaccessible fraction of tested formulations was evaluated in a prostatic epithelium in vitro model, based on tumoral prostatic cells (LNCaP). The prostate-specific anti-inflammatory activity was evaluated pre-treating the in vitro model for 2 h with the bioaccessible fractions corresponding to SRO concentrations reported in Table 8, and then exposing the model to inflamed conditions for 4 h. In particular, prostatic epithelium in vitro model was exposed either to normal monocytic/macrophage cell culture medium (uninflamed condition) or THP-1 cell culture conditioned medium (CM, inflamed condition).

CM was obtained by stimulating overnight PMA-differentiated THP-1 cells with the pro-inflammatory compound LPS (1 ng/mL). LNCaP cells in normal monocytic/macrophage cell culture medium and CM were used as negative and positive controls of inflammation respectively. Diclofenac, a well-known anti-inflammatory drug, was used as a positive control of anti-inflammatory activity. A dose-response curve was performed with MTS assay to determine Diclofenac highest non-lethal concentration on prostatic epithelium in vitro model after 6 h exposure. Similarly, the impact of bioaccessible fractions on LNCaP viability was checked by using MTS assay and morphology. At the end of the treatment, in vitro prostate epithelia were washed with pre-warmed DPBS and detached in ice-cold PBS with a cell scraper. Following centrifugation (1000× *g* for 5 min), LNCaP cells were resuspendend in lysis buffer (protease inhibitor cocktail and 0.1% Triton X-100 in deionized water) and sonicated (5 s pulse-on at 10% amplitude and 25 sec pulse-off, total time 1.5 min) (Sonicator Q700, QSonica, Newtown, CT, USA). Finally, cell lysates were centrifuged for 15 min at 10,000× *g* and the surnatants recovered. Anti-inflammatory activity was determined by measuring the amount of pro-inflammatory cytokines interleukin-1beta (IL-1β) and tumor necrosis factor-alpha (TNF-α) present in cell lysates. IL-1β and TNF-α were quantified by commercial ELISA (Enzyme-Linked Immunosorbent Assay) kits, following the manufacturer’s instructions.

Anti-inflammatory effect of bioaccessible fractions was also evaluated in cell lysate by measuring cyclooxygenase (COX) enzyme 1 and 2 activity. Total COX and COX-2 activities were assessed with a commercial COX activity assay kit, following the manufacturer’s instructions.

##### 3.2.9.1. SRO Pro-Apoptotic Activity

To evaluate the pro-apoptotic activity of the bioaccessible fractions of SRO, a fluorimetric assay, based on caspases 3/7 activation, was performed (Apo-ONE^®^ Homogeneous Caspase-3/7 Assay). This assay is based on the ability of activated caspases 3 and 7 to selectively cleave a specific substrate, making it fluorescent (excitation wavelenght 499 nm, emission wavelength 521 nm). Consequently, the produced fluorescence intensity is linked to the activation of the apoptotic process by cells. To correlate anti-inflammatory with pro-apoptotic activity, the same experimental setup described above for the anti-inflammatory activity was applied. Experiments were performed in triplicate and the assay conducted following manufacturer’s instructions.

##### 3.2.9.2. Smooth Muscle Myorelaxing Activity

The myorelaxing activity of the bioaccessible fraction of tested formulations was evaluated in an in vitro model of smooth muscles, based on WPMY-1 human myofibroblast stromal cell. The myorelaxing effect was analyzed by means of a two-step gel-contraction assay, following the manufacturer’s instructions. The gel contraction assay is characterized by two phases: mechanical stress-generating phase and floating phase. During the first phase, a mix of myofibroblast and collagen is seeded and left to develop mechanical stress for two days while, during the floating phase, gels are released and allowed to freely contract, dissipating the mechanical stress generated during the first phase. Fibroblast-containing gels were exposed to the bioaccessible fraction of the formulations for the duration of the experiment (24 h). The myorelaxation activity was calculated with the following formula:

Myorelaxing Activity (%) = 100 − ((C_exposed_/C_control_) × 100) (2)

where C_fibroblast_ is the contraction of the fibroblast-containing gels exposed to the bioaccessible fraction of the different formulations and C_control_ the contraction of the untreated fibroblast-containing gel (positive control of contraction).

Gel contraction is calculated as follows:

Contraction (%) = 100 − ((D_fibroblast_/D_collagen_) × 100) (3)

where D_fibroblast_ represents the diameter of the fibroblast-containing gel and D_collagen_ the diameter of the collagen gel in which fibroblast were not seeded (negative control of contraction). Experimental and control cells were plated in triplicate and the diameter of each collagen gel was photographed and measurement via images analysis, performed with ImageJ (University of Wisconsin-Madison, Madison, WI, USA).

##### 3.2.9.3. Measurement of Prostate Specific Antigen (PSA) Secretion by LNCaP Prostatic Cells

The effect of the different formulation bioaccessible fraction on the secretion of the androgen-induced prostate-specific antigen (PSA), commonly used as a marker for prostate tumors, was evaluated in LNCaP prostatic cells. After seeding and adhesion (48 h), normal cell culture medium was replaced with a medium containing low hormone level for 24 h (10% charcoal-stripped FBS and 1% Penicillin-Streptomycin mix in RPMI without phenol red). The pre-treatment medium was then removed and cells were incubated with the bioaccessible fraction of each formulation. A control was included in the assay by treating cells with low-hormones cell culture medium. Additionally, control and cells treated with LBF bioaccessible fraction were stimulated with dihydrotestosterone (DHT) (10 nM), known to stimulate the release of PSA [38]. After 24 h exposure, media were collected for measurement of secreted PSA using a commercial ELISA kit (Abcam), following the manufacturer’s instructions. Results were expressed as percentage of secreted PSA in cells treated with different formulations compared to control.

### 3.3. Statistical Analysis

Results from performed experiments were statistically analyzed using OriginLab (OriginLab Corporation, Northampton, MA, USA) software. Experiments were performed in triplicate, results presented as average ± standard deviation. A *p* value of ≤0.05 was considered significant.

## 4. Conclusions

In the present paper, we demonstrated the successful application of a new technological lipid-based delivery matrix (Lipomatrix) able to protect lipophilic plant-derived active principles from the gastric compartment and favoring the formation of hydro-dispersible forms in the duodenum, thanks to the pH-dependent ionization of ASP. Lipomatrix-based formulation, despite the not improved apparent bioaccessibility data, seems to enhance overall enteric absorption of SRO in a CACO-2 cells model and this data well correlates with an overall improvement of its biologic effects on in vitro prostatic tissue model, compared to not formulated commercial SRO. Indeed, we showed that the Lipomatrix-associated fraction of SRO reduced inflammation and modulated cytokines pattern expression, enhanced pro-apoptotic caspases-mediated activity and improved myo-relaxation significantly better than not formulated SRO contained in the mentioned commercial products. Lipomatrix-associated SRO shoved furthermore to significantly reduce PSA expression in *LNCaP* prostatic cells but not better than one of the two SRO-containing commercial formulations tested. Moreover, even though as preliminary evidence, SRO formulated in Lipomatrix seems to ensure a higher safety profile on in vitro intestinal model in terms of overall cell vitality, compared to the tested commercial formulations. 

To conclude, taken together the data collected in this work, it is possible to consider Lipomatrix as a promising, next generation technological platform for improving enteric delivery of SRO and as consequence its efficacy on human prostatic gland, so confirming the potential significant role of delivery systems in bioavailability of natural lipophilic compounds. 

## Figures and Tables

**Table 1:** Cristallinity index, entropy, and extrapolation pick time of Lipomatrix and any of its constituents.

Cryst. Ind./Entropy/Extr. P	MDGFA	Mannitol	ASP	SRO	LIPOMATRIX with SRO
Crystallinity Index (CI) (%)	15.75	12.50	11.49	n.r.	12.99
Entropy (mJ)	−529.19	−2122.40	−837.90	n.r.	−705.02
Exprapol. Peak (min)	1.74	7.17	4.52	n.r.	7.20

**Table 2:** Principal parameters of particle size distribution of the LIPOMATRIX in FaSSIF-V2 sample after sonication.

Sample Name	Hydrodynamic Diameter (Average ± Standard Deviation, nm)	Polydispersity Index
FaSSIF-V2	769 ± 274 161 ± 55 5468 ± 248	0.703
LIPOMATRIX in FaSSIF-V2	528 ± 378 27 ± 6 4511 ± 881	0.529

**Table 3:** Total amount and apparent bioaccessible fraction of SRO for the three tested formulations, expressed as a percentage of the total SRO in a single dose (*n* = 3).

*Serenoa Repens* (Fatty Acids)			
Formulation	Total Amount Post-Digestion (%)	Apparent Bioaccessible Fraction (%)	Emulsifying Efficiency (%)
LBF	54.0	27.7 ± 3.7	51.3
CF1	62.4	30.1 ± 4.6	48.2
CF2	57.2	29.8 ± 7.4	52.0

**Table 4:** Total amount and bioaccessible fraction of PCA and LYC, expressed as a percentage of the total Flowens^TM^ and total lycopene in a single dose of LBF. (*n* = 3).

	Total Amount Post-Digestion (%)	Apparent Bioaccessibility (%)
Proanthocyanidins	100	98.6
Lycopene	100	62.5

**Table 5:** Half maximal effective concentrations (EC_50_) from dose-response curves.

Formulation	EC_50_ (mg/mL)
LBF	8.8 ± 2.1
CF1	2.1 ± 0.1
CF2	7.8 ± 0.7

**Table 6:** SRO absorption rate expressed as a percentage of absorption ± standard error (SE) (*n* = 3).

Formulation	*S. repens* Absorption Rate (% ± SE)
LBF	22.0 ± 12.7
CF1	18.8 ± 12.2
CF2	3.2 ± 3.2

**Table 7:** List and composition of analyzed formulations.

Formulation	Active Composition	mg/capsule
Lipomatrix-based formulation (LBF)	*S. repens* (Bartram) small oil (85% fatty acids, GC)	160
	Flowens^TM^	125
	Lycopene (6% minimum)	5
Commercial formulation 1 (CF1)	*S. repens* (Bartram) small oil extract from fruit	320
Commercial formulation 2 (CF2)	*S. repens* (Bartram) small oil extract from fruit (88% fatty acids)	320
	*Pinus massoniana* L. dry extract from bark (95% proanthocyanidin)	120
	*Crocus sativus* L. dry extract from stigmas (0.3% safranal)	100

**Table 8:** Absorption rate expressed as a percentage of SRO absorption ± standard error (SE) and concentration (µg/mL) at the basolateral compartment (serosal).

Formulation	*S. Repens* Absorption Rate (% ± SE)	*S. Repens* Concentration in the Serosal Compartment (µg/mL)
Lipomatrix	22.0 ± 12.7	300
CF1	18.8 ± 12.2	270
CF2	3.2 ± 3.2	50

## Supplementary Materials

Supplementary materials can be found at http://www.mdpi.com/1422-0067/20/3/669/s1.
