# Supplementary material for: Lipomatrix: A Novel Ascorbyl Palmitate-Based Lipid Matrix to Enhancing Enteric Absorption of Serenoa Repens Oil
Source: Int J Mol Sci. 2019 Feb 4;20(3):669. doi: 10.3390/ijms20030669 (PMC6386960; doi:10.3390/ijms20030669)
Supplement: Supplementary file 1 [file ijms-20-00669-s001.pdf]

SUPPLEMENTARY FILE

Lipomatrix: a novel Ascorbyl Palmitate-based lipid matrix to enhancing enteric absorption of Serenoa Repens Oil

A. Fratter[a,b], V. Mason[a], M. Pellizzato[a], S. Valier[c], A.F.G. Cicero[d], Erik Tedesco[e], Elisa Meneghetti[e], F. Benetti[e]

[a]Nutraceutical Research and Innovation Technology Department, Labomar Research, Istrana (TV), Italy

[b]Head, Nutraceutical Research and Innovation Technology Department, Labomar Research, Istrana (TV), Italy

[c] PHF SA, 6900 Lugano, Switzerland

[d] University of Bologna, Faculty of Medicine and Surgery, Department of Cardiovascular disease prevention research unit, Internal Medicine and lipidology, Italy;

[e] ECSIN-European Center for the Sustainable Impact of Nanotechnology, ECAMRICERT SRL, I-45100 Rovigo, Italy

Corresponding author:

E-mail address: andrea.fratter@labomar.com (A. Fratter)

Via Fabio Filzi, 55, 31036, Istrana (TV), Italy

Ascorbyl Palmitate physical and chemical properties

The main antioxidant and redox property of ASP, the same of Ascorbic acid (AA), is strictly connected to the low value of the standard redox potential (E0) of ascorbic head according to the Nerst's equation in the following reaction:

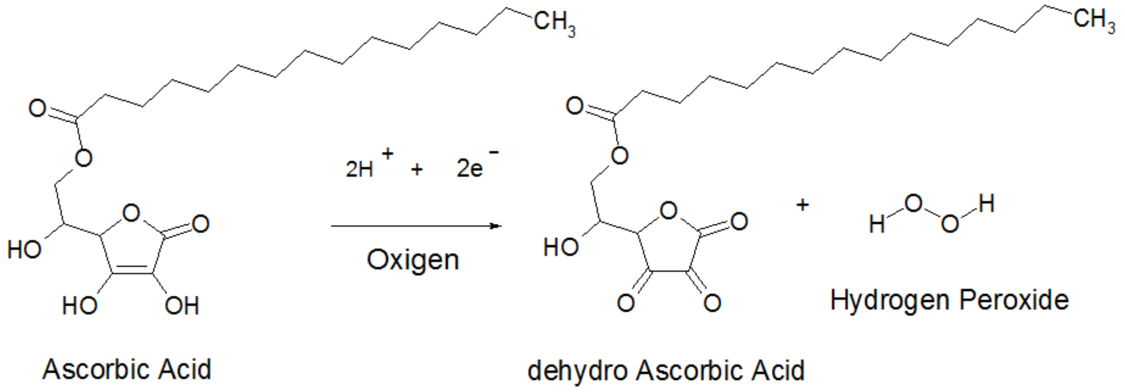

$E=E^0+RT/nF \times Log[Red]^o/[Ox]^p$ (Nerst's equation)

Where:

E= Energy exchanged in the system

E^0=Redox standard potential

F=Faraday constant

R=Gas constant (8,314 J mol^{-1} K^{-1})

T= Absolute temperature

n= Number of electrons exchanged in the reaction

[Red]= Concentration of the reduced species

[Ox]= Concentration of the oxidized species

o,p=Stoichiometric coefficient of the reaction

ASP is a fat-soluble molecule that, thanks to the saturated chain of palmitic acid and despite the ascorbic hydrophilic head, fully dissolves in the most part of oils and melted glycerides, even at relatively high percentage w/w, by warming up the phase. On the other side, ASP is an amphiphilic molecule, for the same aforementioned reasons and can behave as an emulsifying agent under certain conditions. More precisely, the enediolic moiety expressed

in the ascorbic head of ASP can be ionized in presence of an alkali such as alkaline or alkaline-hearth metals hydrates (sodium or calcium hydrate) or alkaline aminoacids such as L-Arginine in water solution (**Figure 1**) taking place to a yellowish, viscous, clear system.

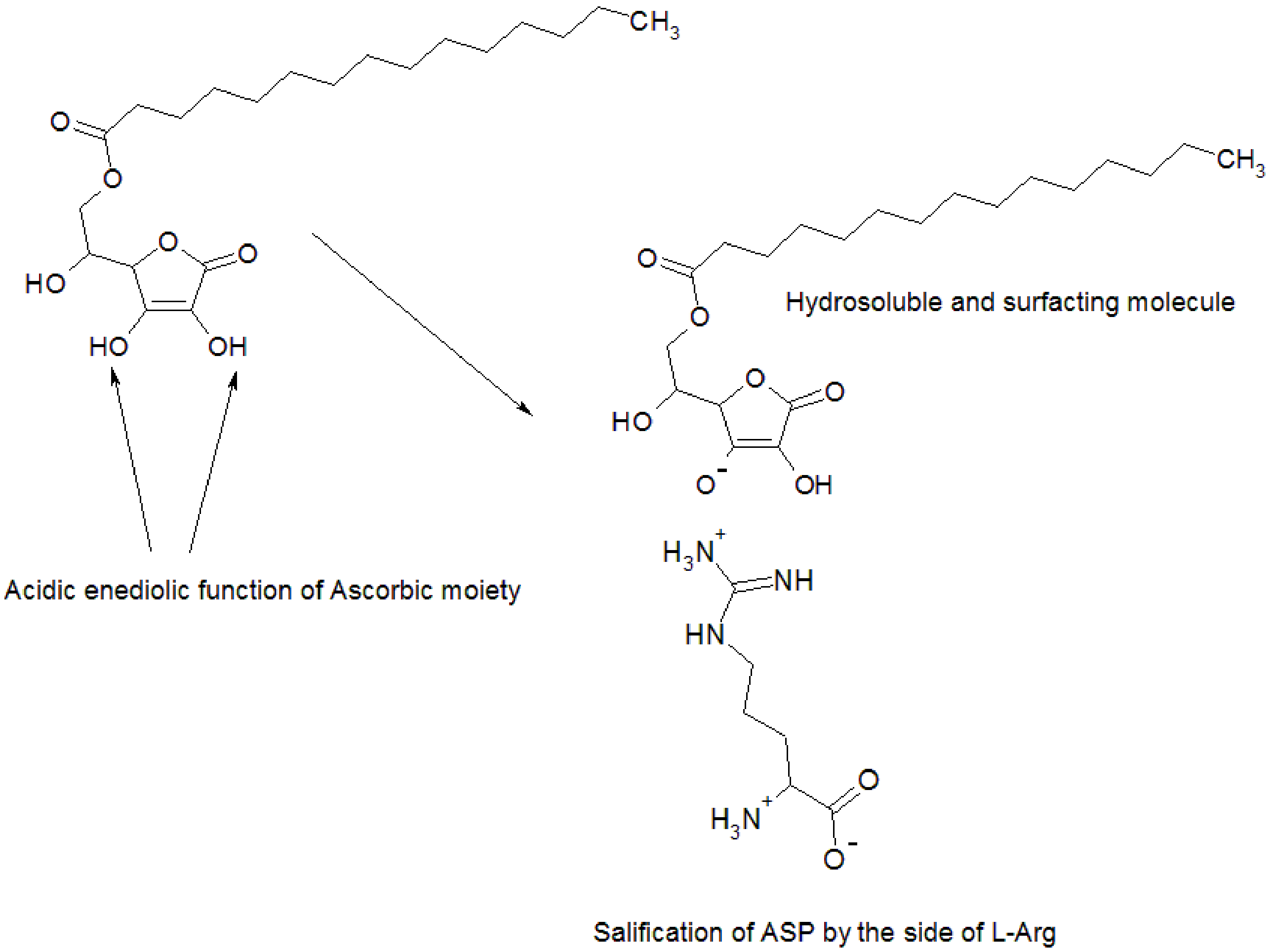

Figure 1. *Salification of ASP in presence of alkali.*

A clear solution of ASP at 5-6% w/w can be achieved warming up the phase, but after cooling, the progressive dissipation of the thermal energy produces a less moveable isotropic liquid structure with the occurring of an ordinate assembling of the ASP molecules that takes place to a highly structured hard gel (coagel) (***Figure 2***).

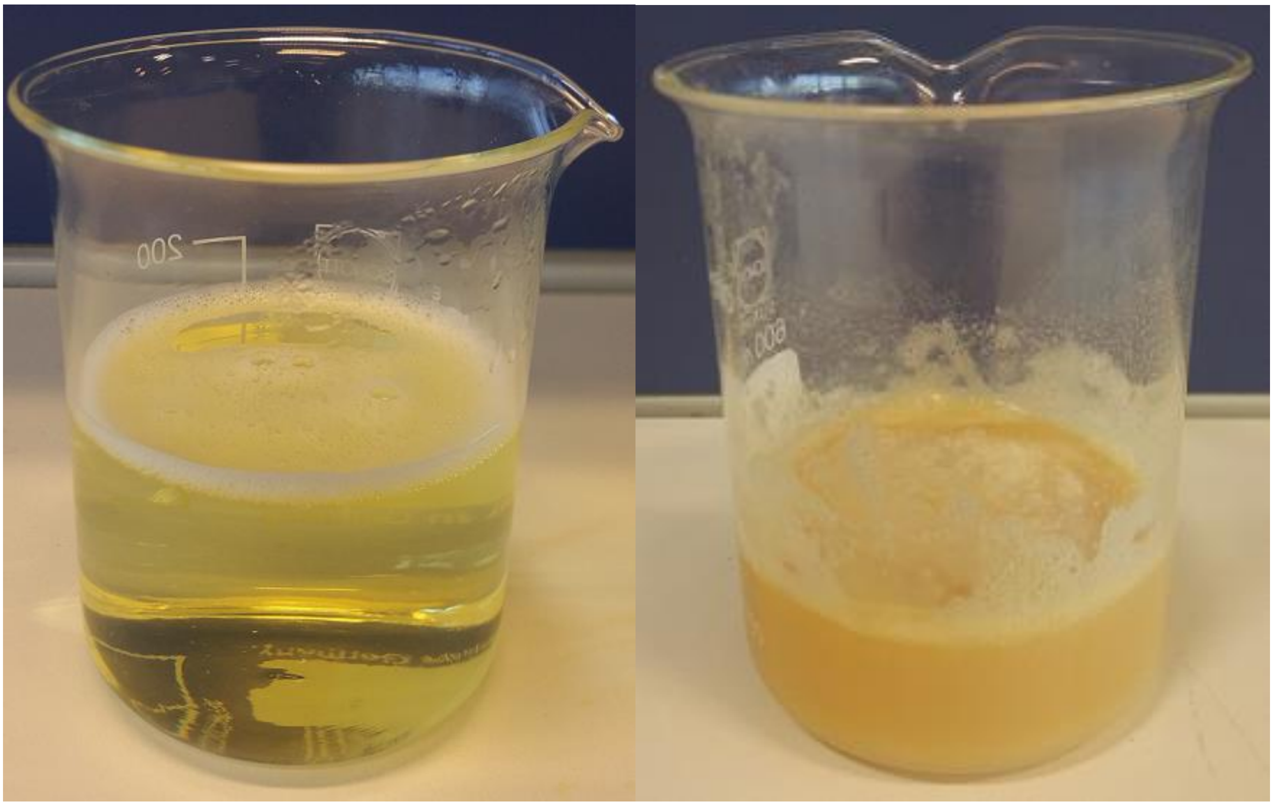

Figure 2. *(left) Solubilization of ASP in water at 5% w/w in presence of sodium hydrate 0,2 N (pH=8) at 60°C. (right) After cooling, the system appears as a semi-solidified coagel: the dissipation of thermal energy induces the formation of a highly packaged assembling structure.*

ASP has been deeply studied as potential emulsifying-solubilizing agent able of creating peculiar micellar structures in which poorly soluble molecules of pharmaceutical interest, such as Griseofulvin, can be usefully solubilized (Aspasomes^{TM}) [1]. The peculiar shapes of ASP micellar structures that occurring in water medium depending on ASP concentration, heating and pH and the relative chemical and physical properties have been extensively studied and characterized [2,3,4]. The further, not less important advantage of ASP as emulsifying agent of lipophilic compounds, is that it exerts an effective antioxidant and anti-peroxidation activity that can come in handy to maintain integrity of highly oxidizable molecules such as fish oils, carotenoids, Coenzyme Q10 and other unsaturated molecules, over time and during industrialization [5,6].

DSC thermograms

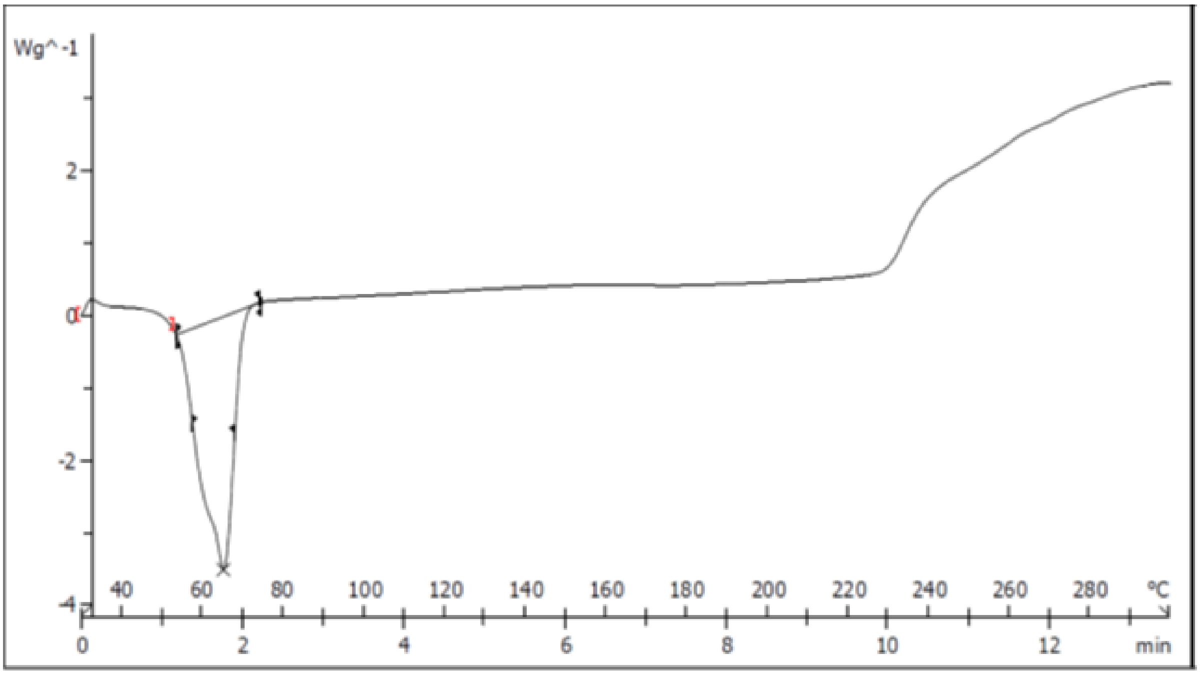

Graph 1. Thermogram of MDGFA. It is recognizable an endothermic melting pick between 50 and 70°C according to the melting point of the raw material (57°C).

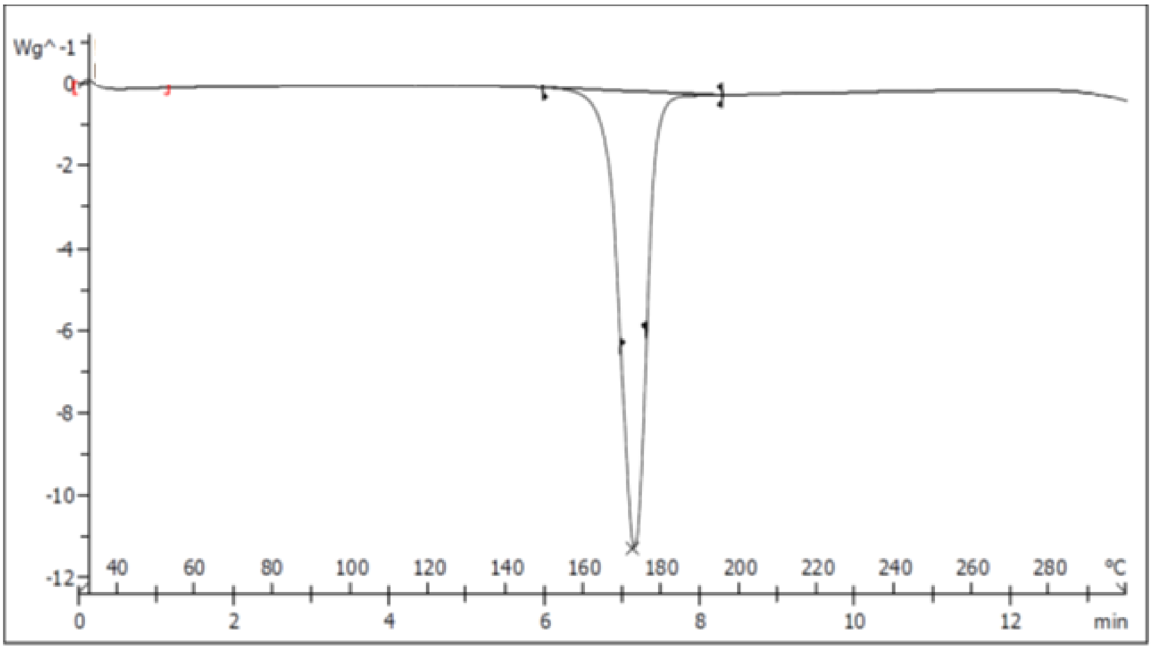

Graph 2. Thermogram of Mannitol. it shows an endothermic fusion pick at 170°C, data according to the technical data sheet of the raw material for melting point.

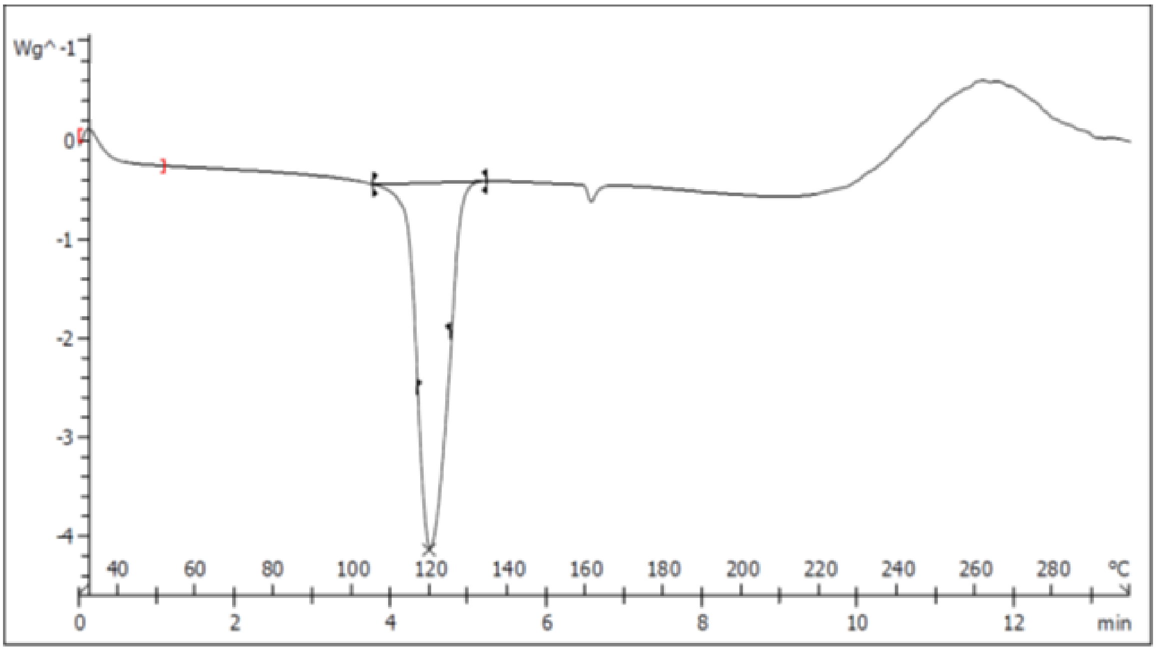

Graph 3. Thermogram of ASP. It melts showing an endothermic pick around 115-120°C and an exothermic pick at 260° C.

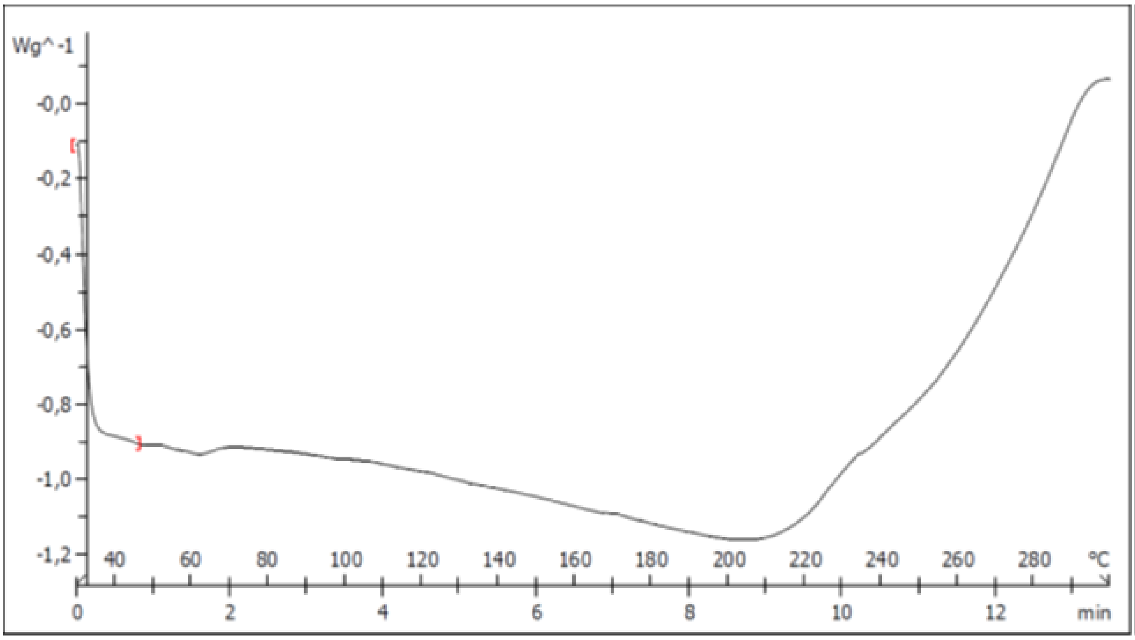

Graph 4. Thermogram of SRO (30-300°C). The thermogram shows an exothermic large pick after 210°C, likely corresponding to the oil degradation; it was not possible to investigate the typical exothermic crystallization behavior of a vegetable oil, because the device employed was not equipped with an intracooler.

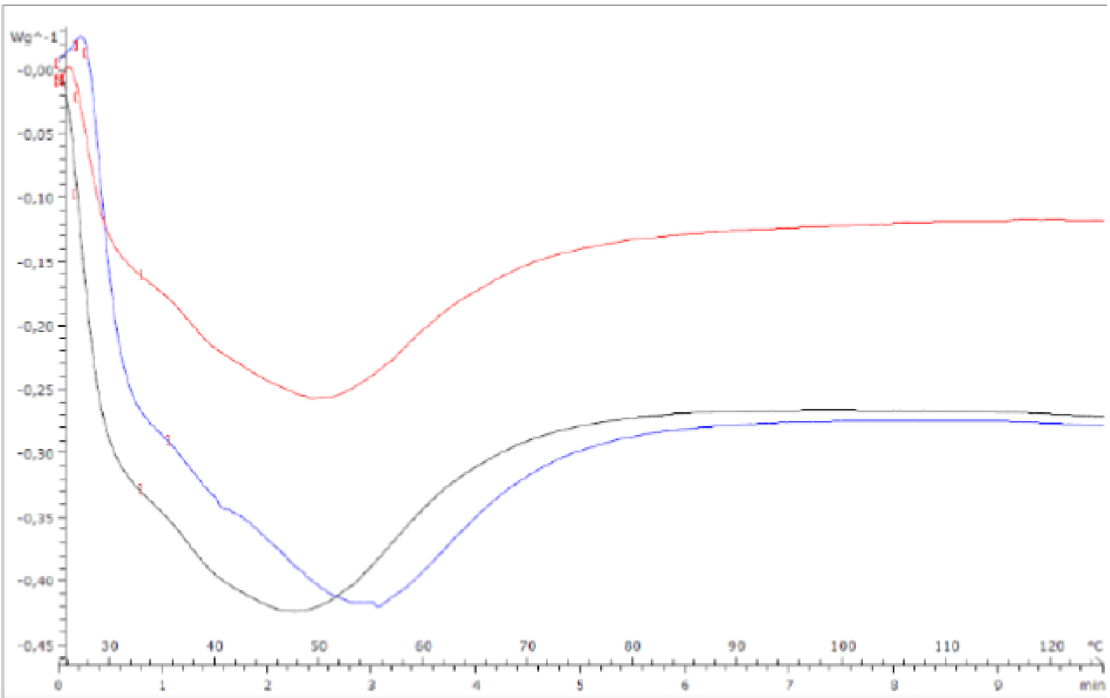

Graph 5. Thermogram of Lipomatrix in the range 25-125°C.

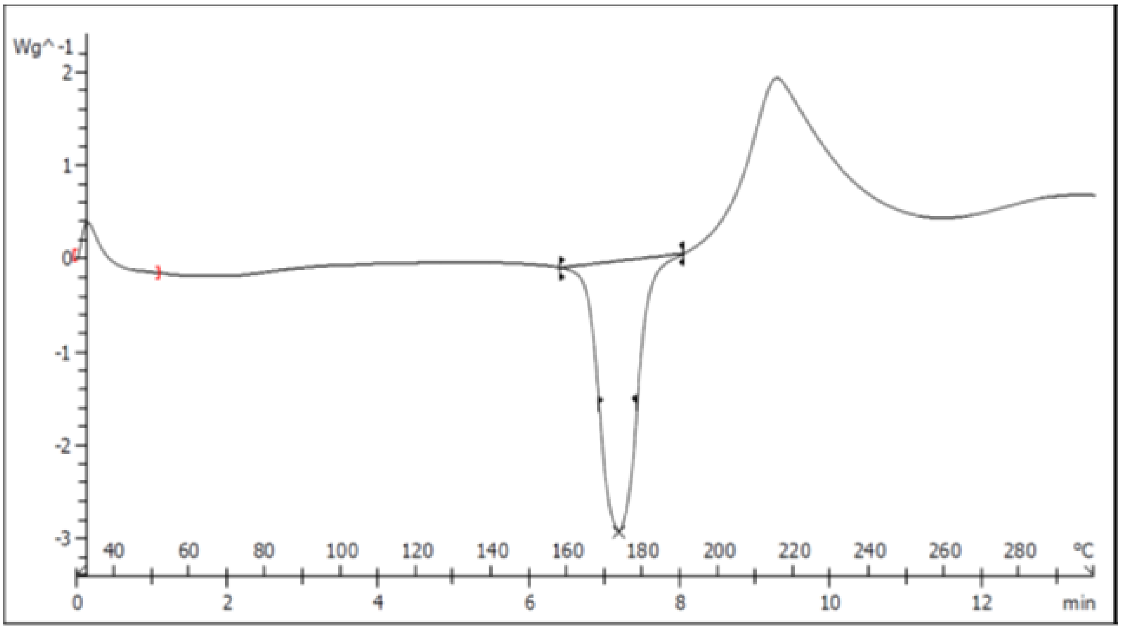

Graph 6. Thermogram of Lipomatrix with SRO in the range 30-300°C. It is recognizable a melting phenomenon with an endothermic pick at 170°C, very close to Mannitol one and an exothermic pick between 200 and 240°C.

GC-MS graphs relating to GSF aliquots containing Lipomatrix with SRO

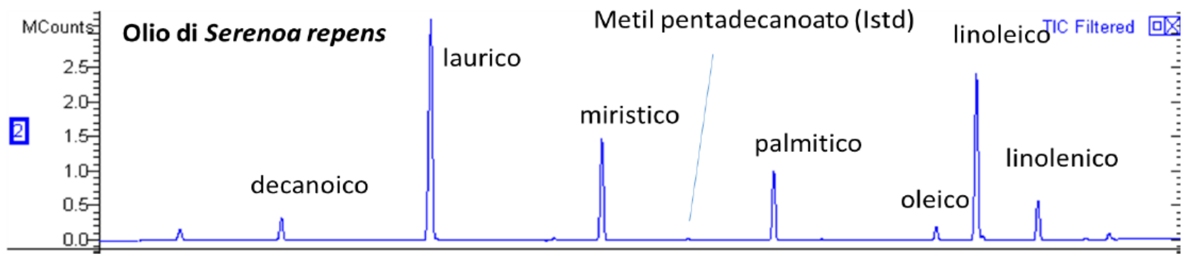

Graph 1. *GC-MS spectrum of SRO raw material employed for the described experiments. The pick relating to the main fatty acids constituents are recognizable.*

Lipomatrix Test 01

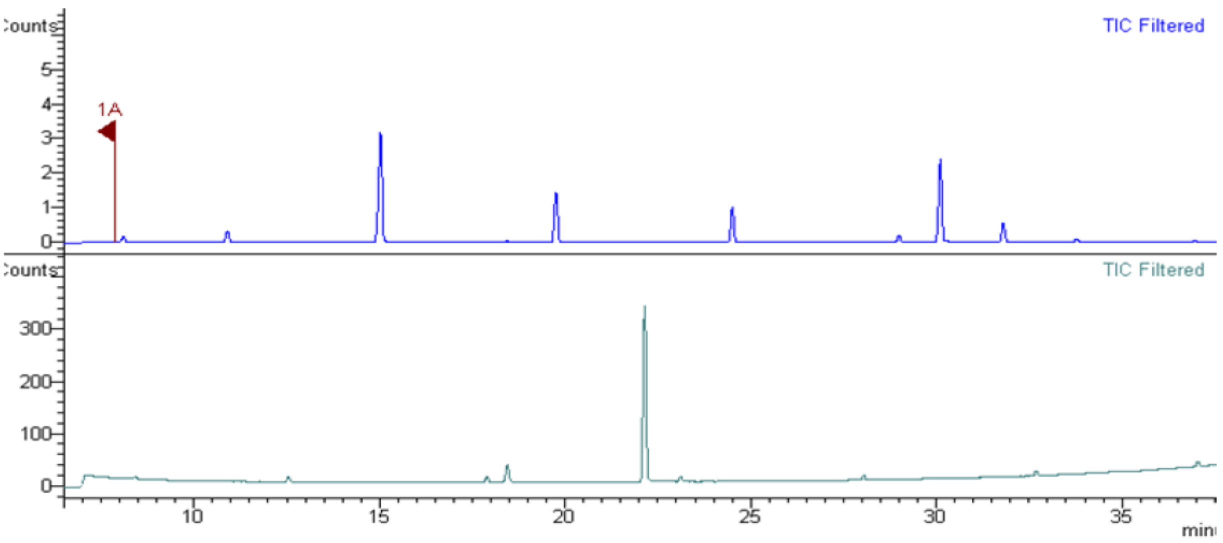

Graph 2. *GC-MS spectrum of the first aliquot coming from disaggregation test of Lipomatrix in GSF (triplicate analysis).*

Lipomatrix Test 02

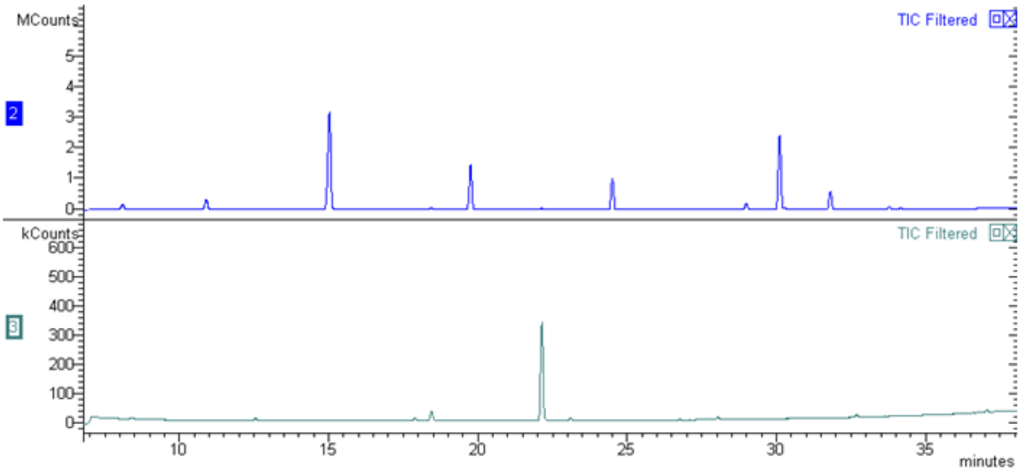

Graph 3. *GC-MS spectrum of the second aliquot coming from disaggregation test of Lipomatrix in GSF (triplicate analysis).*

Lipomatrix Test 03

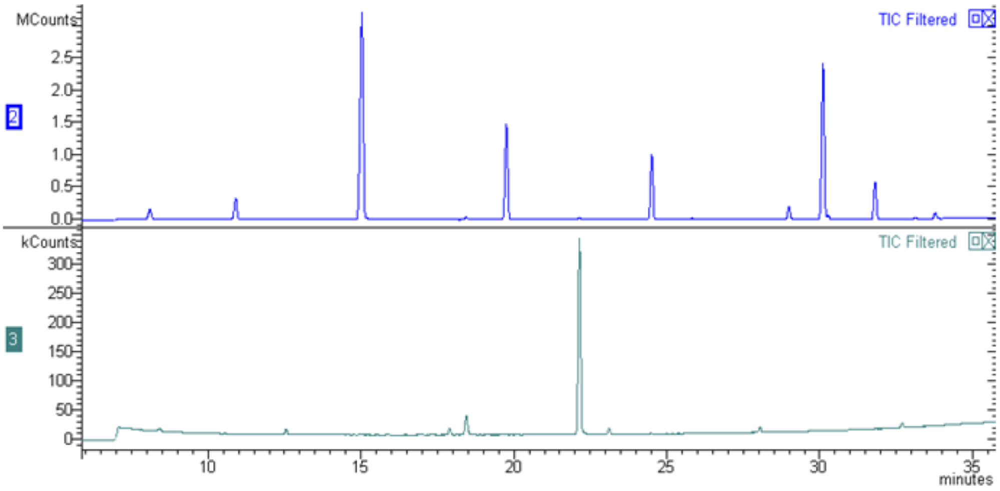

Graph 4. *GC-MS spectrum of the third aliquot coming from disaggregation test of Lipomatrix in GSF (triplicate analysis).*

Bibliography

1. Gopinath D et al. Ascorbyl palmitate vesicles (Aspasomes): formation, characterization and applications. Int J Pharm. 2004 Mar 1;271(1-2):95-113.
2. Palma S et al. Evaluation of the surfactant properties of ascorbyl palmitate sodium salt. Eur J Pharm Sci. 2002 Jul;16(1-2):37-43.
3. Palma S et al. Nanostructures from alkyl vitamin C derivatives (ASCn): properties and potential platform for drug delivery. Int J Pharm. 2007 Dec 10;345(1-2):26-34.
4. Palma S et al. Coagels from alkanoyl-6-O-ascorbic acid derivatives as drug carriers: structure and rheology. Farmaco. 2003 Dec; 58(12):1271-6.
5. Zou L, Akoh CC. Oxidative stability of structured lipid-based infant formula emulsion: effect of antioxidants. Food Chem. 2015 Jul 1;178:1-9. Food Chem. 2015 Jul 1;178:1-9.
6. Yuwei Liu, Zhanqun Hou, Jia Yang, and Yanxiang Gao. Effects of antioxidants on the stability of β-Carotene in O/W emulsions stabilized by Gum Arabic. J Food Sci Technol. 2015 Jun; 52(6): 3300–3311.
